# Chamomile: A Review of Its Traditional Uses, Chemical Constituents, Pharmacological Activities and Quality Control Studies

**DOI:** 10.3390/molecules28010133

**Published:** 2022-12-23

**Authors:** Yun-Lei Dai, Ying Li, Qi Wang, Feng-Jv Niu, Kun-Wei Li, Yun-Yu Wang, Jian Wang, Chang-Zheng Zhou, Li-Na Gao

**Affiliations:** College of Pharmacy, Shandong University of Traditional Chinese Medicine, Ji’nan 250355, China

**Keywords:** *Matricaria chamomilia* L., *Chamaemelum nobile* (L.) all., chamomile, chemical constituents, pharmacological activities

## Abstract

*Matricaria chamomilla* L. (MC) and *Chamaemelum nobile* (L.) All. (CN) are two varieties of Chamomile. These herbs have been used for thousands of years in Greece, Rome and ancient Egypt. Chamomile has been used for the treatment of stomach problems, cramps, dermatitis, and minor infections. The purpose of this study was to introduce the botanical characteristics and geographical distribution, traditional uses, chemical constituents, pharmacological activities, toxicity studies and quality control studies, and lay a theoretical foundation for the rational development and utilization of chamomile. This review powered that chemical constituents include flavonoids, coumarins, volatile oils, terpenes, organic acids, polysaccharides, and others. These compounds possess anticancer, anti-infective, anti-inflammatory, antithrombotic, antioxidant, hypolipidaemic, hypoglycaemic, antihypertensive, antidepressant, neuroprotective activities, among others. Chamomile is a widely used herb in traditional medicine. It brings great economic value due to its numerous pharmacological effects and traditional uses. However, more toxicity tests should be carried out to confirm its safety. There is need for further research to provide concrete scientific evidence and validate its medicinal properties.

## 1. Introduction

Traditional Chinese medicine (TCM) has been used for a long time in China and recorded for more than 5000 years. Numerous herbs have been used in TCM [1]. This field is becoming an integral part of various traditional medicine and modern medicine systems globally. In modern medicine, it is primarily used to prevent various diseases. Therefore, TCM is gaining popularity worldwide and supports human health greatly [2].

Chamomile is an annual or perennial plant belonging to the family Asteraceae. The plant improves the appetite and relieves painful swellings and sweating [3]. Chamomile is native to temperate regions of Asia and Europe, and cultivated worldwide for its high medicinal, cosmetics and food value [4]. It has been used for thousands of years in Greece, Rome and ancient Egypt. In China, the detailed use of this plant was first recorded in Uyghur medicine. Veteran doctors of TCM believe that preparations containing chamomile have a calming effect. In addition, the plant is also used in other traditional, homoeopathic and Unani preparations [5,6]. There are two main varieties of chamomile: *Matricaria chamomilia* L. (MC) and *Anthemis nobilis* (L.) All. (CN). *Matricaria chamomilia* L. belongs to the genus *Matricaria*. It is an annual plant, and the flowering period is from May to July in China. *Chamaemelum nobile* (L.) All. is a perennial plant of the genus *Chamaemelum*. The flowering period is from April to May in China [7,8]. *Matricaria chamomilia* L. is relatively common and has been researched and used widely. At present, 26 countries around the world have included this plant in their pharmacopoeia. Chamomile flower heads are commonly used for medicinal purposes [9]. Chamomile contains flavonoids, coumarins, volatile oils, terpenes, sterols, organic acids, and polysaccharides, among other compounds. Having a wide array of compounds, chamomile exhibits various pharmacological activities such as anticancer, anti-infective, anti-inflammatory, antioxidant, hypoglycaemic, hypotensive, hypolipidaemic, antiallergic, antidepressant, and neuroprotective effects, and others [3,4,5,6,7,8]. In general, this plant has outstanding research value. Still, there are very few reviews of it in the literature. This article provides a comprehensive review of the botanical characteristics and distribution, traditional uses, chemical constituents, pharmacological effects, and quality control methods of chamomile.

**Figure 1:**
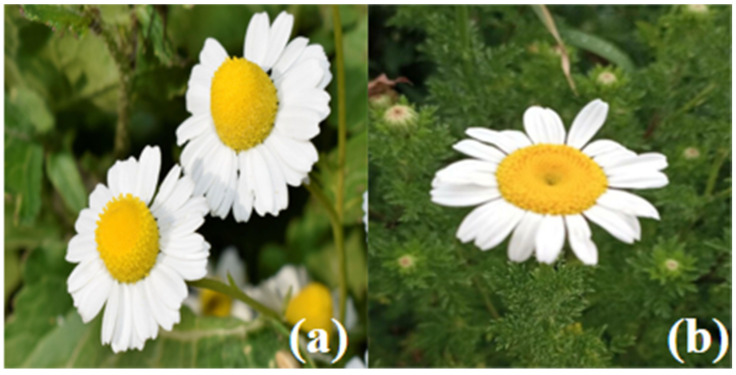
Two species of Chamomilla: *Matricaria chamomilia* L. (**a**), *Chamaemelum nobile* (L.) All. (**b**).

Pictures of two species of chamomile from the Global Biodiversity Information Facility “https://www.gbif.org (accessed on 16 November 2022)” are shown in Figure 1.

## 2. Methodology

In this review, the information about the botanical characteristics and geographical distribution, traditional uses, chemical constituents, pharmacological activities, adverse reactions, toxicity studies and quality control studies of chamomile is collected. All information on chamomile and its two species were collected from textbooks, electronic databases and library materials, including PubMed, Web of Science, China National Knowledge Infrastructure, MDPI, Springer, Elsevier, Google Scholar, Yahoo search and Google Scholar. The keywords used to obtain the information. included ‘Yang Gan Jv’, ‘Chamomile’, ‘*Matricaria chamomilia* L.’, ‘*Chamaemelum nobile* (L.) All.’, ‘botany’, ‘traditional uses’, ‘pharmacological activities’, ‘chemical constituents’, ‘toxicity’, and ‘quality control studies.’ The scientific names and photos of two species of chamomile were confirmed from the World Flora Online database (www.worldfloraonline.org). The chemical constituents were verified using PubChem, and the structures were drawn using ChemDraw.

### 2.1. Botanical Characteristics and Geographical Distribution

Chamomile is an annual or perennial plant native to temperate regions of Asia and Europe. It is widely cultivated worldwide, such as in Germany, Hungary, France, Russia, Brazil and western Xinjiang of China. This plant is native to tropical conditions but it can be cultivated in cold climatic conditions [4]. The roots are thin, spindle-shaped and grow straight. The stems can grow to 10–80 cm. The leaves are long, narrow and pinnate, with fissures. The head is about 10–30 mm in diameter [5,10]. *Matricaria chamomilia* L. has small white flowers of the Compositae family and, at their center, a yellow tubular petal is present. *Anthemis nobilis* (L.) All. contains flowers with double petals, soft stems, and has a green apple fragrance. It is also called “the apple of the ground”. In addition, it is also known as the “Physician of Plants” due to its ability to heal sick plants around it. Although MC and CN are similar in appearance, they have distinct differences. The petals of MC are turned down and have a raised conical center, whereas the center of CN is flat [8]. Additionally, MC and CN have four and three germination holes in pollen grains, respectively. It is worth noting that CN has a non-glandular hair structure [6,11]. In some areas, such as Europe, Mexico, South America, Russia, the chamomile flower head is the main medicinal part and is used for anti-inflammation and sedation. However, in China, in the majority of cases the whole plant is collected to treat various diseases [7].

### 2.2. Traditional Uses

As early as the Eastern Han Dynasty in China, a monograph recorded the use of TCM by human beings to treat sundry diseases, namely “Shennong’s Classic of Materia Medica” (《神农本草经》) [12]. Several uses of chamomile are reported in the TCM literature, and it is one of the most commonly used herbal medicines to treat stomach problems, cramps, dermatitis, and minor infections [13]. Chamomile has been used for thousands of years in Greece, Rome, and ancient Egypt. In China, the plant was first recorded in detail in Uyghur medicine. In the “Zhu Medical Canon” (《注医典》), a Uyghur medical work written in the 10th century, chamomile is called “Bamu Nai”. Its taste and fragrance is slightly bitter. The plant nourishes the nerves and the stomach. It improves the appetite and relieves painful swellings and sweating, and is frequently used for chronic headaches, constipation, poor sweating, joint swelling, and urinary system disorders [3,5]. “Chinese Herbs. Uyghur Medicine Volume” (《中华草本.维药卷》) recorded that Chamomile can strengthen the brain and muscles, can be a diuretic and improves e menstrual flow, nourishes the stomach and can be used as an appetizer. It mainly treats symptoms of muscle relaxation, joint swelling and pain, stomach deficiency and anorexia, amenorrhea, and urine retention. “Chinese Ethnic Medicine” (《中国民族药志》) indicates that Chamomile can detoxify, clear heat, stop dysentery, dispel wind, and eliminate paralysis. Meanwhile, the Uyghur folk medicine book “Baidi Yi Medicine Book” (《拜地依药书》) recorded that the plant has a favourable effect on cold headache, black fat and mucous typhoid, and kidney stones [6,7]. It is worth mentioning that 26 countries worldwide have included Chamomile in pharmacopoeia, including German Pharmacopoeia, European Pharmacopoeia, United States Pharmacopeia, British Pharmacopoeia, and more [9]. It can be used as an ingredient in many traditional medicine preparations, such as Zukamu granules, Fufang Munizi granules, and Strong Madsiri Ayat honey ointment, and is also used in Homeopathy and Unani preparations [5,7]. Furthermore, chamomile is the main active ingredient in many mouthwash preparations [14]. The oral consumption of this plant relieves pain caused by functional digestive disorders and symptoms of gastrointestinal disorders. Topical application of chamomile essence (as a lotion or powder) can be used to repel mosquitoes, treat skin diseases, wounds, haemorrhoids and inflammation of the eyes, nose, and throat [7,15,16]. It has been used in herbal baths for thousands of years, and is commonly consumed as a decoction (in water) in TCM [17]. According to numerous records, chamomile is one of various TCM bath agents.

In fact, in addition to being used as a medicine, chamomile is also used in cosmetics, food, and more [18,19]. The essential oil in this plant serves as perfume, an in skincare products, massage oil, and toothpaste. [20]. A small amount of chamomile in bathing water results in emollient and anti-inflammatory effects [21]. Tea made from dried flowers is reported to induce good sleep, regulate the intestines and sweat, and prevent cold [18]. Honey chamomile tea brewed with 5 g of Chamomile, two slices of lemon, and one tablespoon of honey has been reported to lower blood pressure, detoxify, and relieve inflammation [22]. Gould et al. found that chamomile tea could influence hemodynamics in patients with heart disease [23]. Guo et al. used the essential oil of CN to prepare a clear and transparent flower tea product with a unique flavor [24]. A medicinal preparation containing chamomile and other herbs produces calming and tranquillizing effects. The traditional uses of chamomile are shown in Table 1.

### 2.3. Chemical Constituents

#### 2.3.1. Organic Acids

Organic acids contain carboxylic acid, and sulfonic acid functional groups. A total of 26 organic acids have been isolated from chamomile, among which four acids are primary metabolites and are essential compounds for the growth and development of living organisms [33,34,35,36,37,38]. The compounds also have great potential in the treatment of cardiovascular diseases, immune system diseases and cancer [39]. In addition, the remaining 22 acids are secondary metabolites.

**Figure 2:**
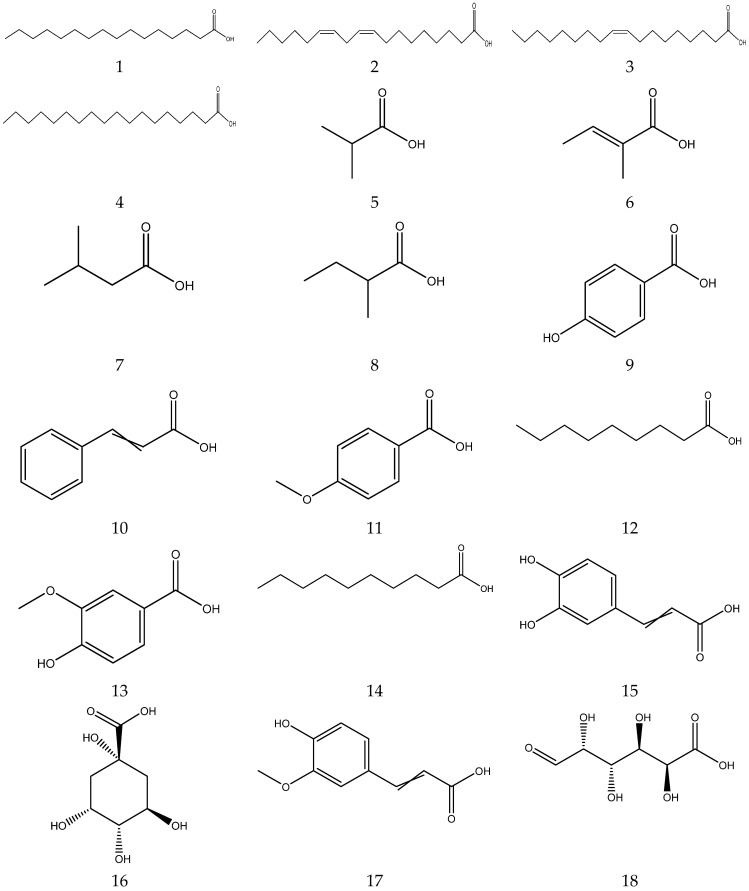

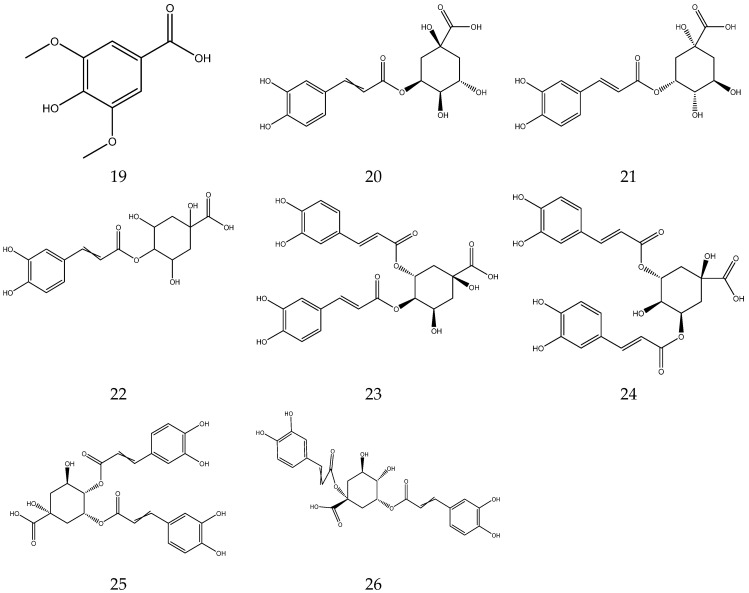
The chemical structures of organic acid from Chamomile.

The organic acids from chamomile are shown in Table 2, and their chemical structures are shown in Figure 2.

#### 2.3.2. Flavonoids

In general, flavonoids have a core structure of 2-phenyl chromone. A total of fifty flavonoids have been isolated from Chamomile and are its main active components [33,34,35,36]. They include quercetin, apigenin, luteolin, and rutin. These compounds exhibit antibacterial, antioxidant, anticancer, and other pharmacological effects. Yang et al. reported the presence of apigenin-7-O-β-D-glucoside and luteolin-7-O-β-D-glucose glycosides in an alcohol extract, and these are assumed to be the main bioactive flavonoids of chamomile [40]. Mire Ayi et al. evaluated the inhibitory effect of a total flavonoid extract on pancreatic lipase using 4-methylumbelliferone oleate (4-MUO) as a substrate [34]. Therefore, chamomile could be an alternative medicine to prevent and treat obesity. According to reports, apigenin has anti-inflammatory properties. Apigenin reduces inflammation in lipopolysaccharide (LPS)-stimulated BV2 microglia via the Glycogen Synthase Kinase 3β/Nuclear factor E2 related factor 2 (GSK-3β/Nrf2) signaling pathway [41]. In addition, it affects the levels of interferon-γ and interleukin-10 in lymphocytes [42].

**Figure 3:**
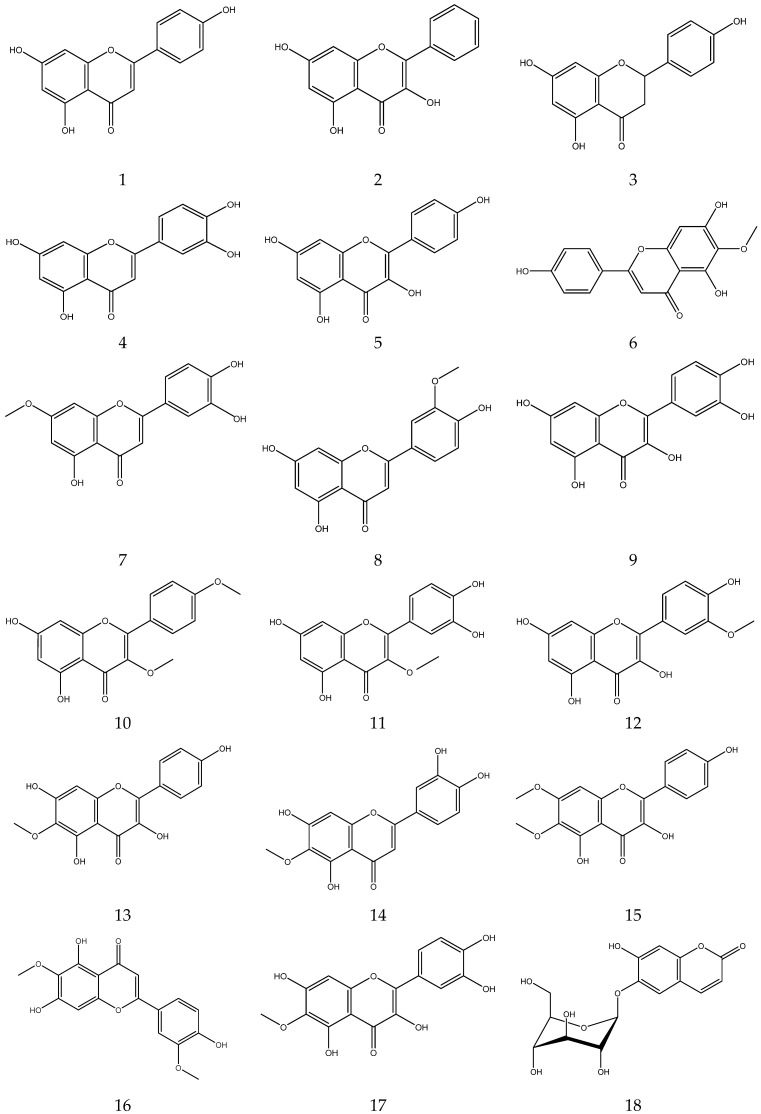

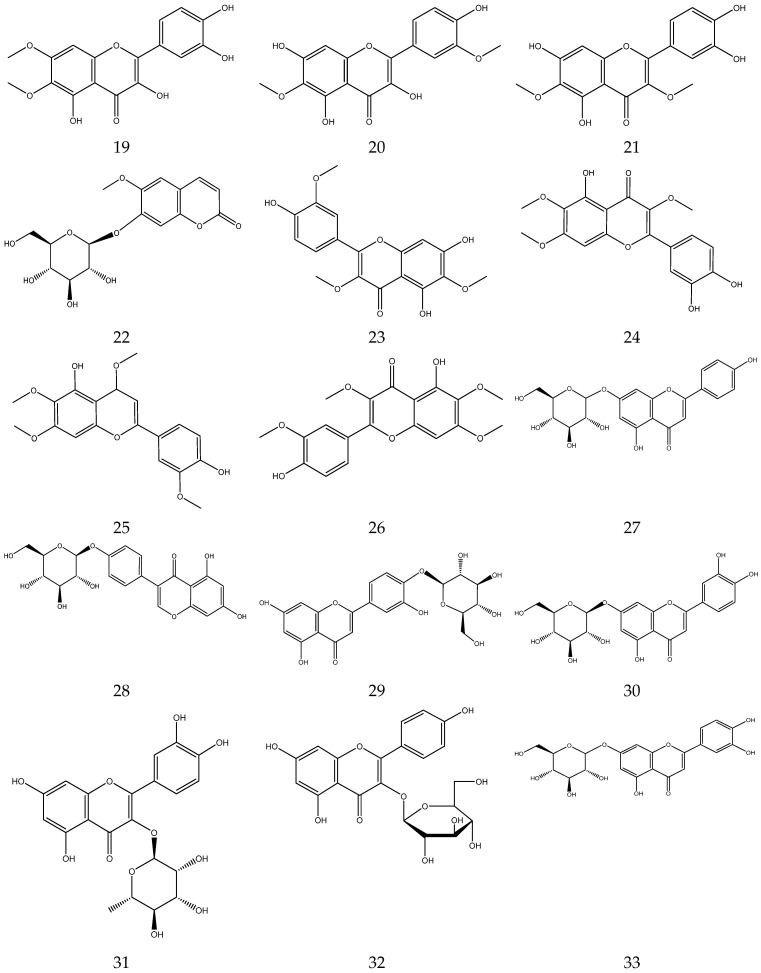

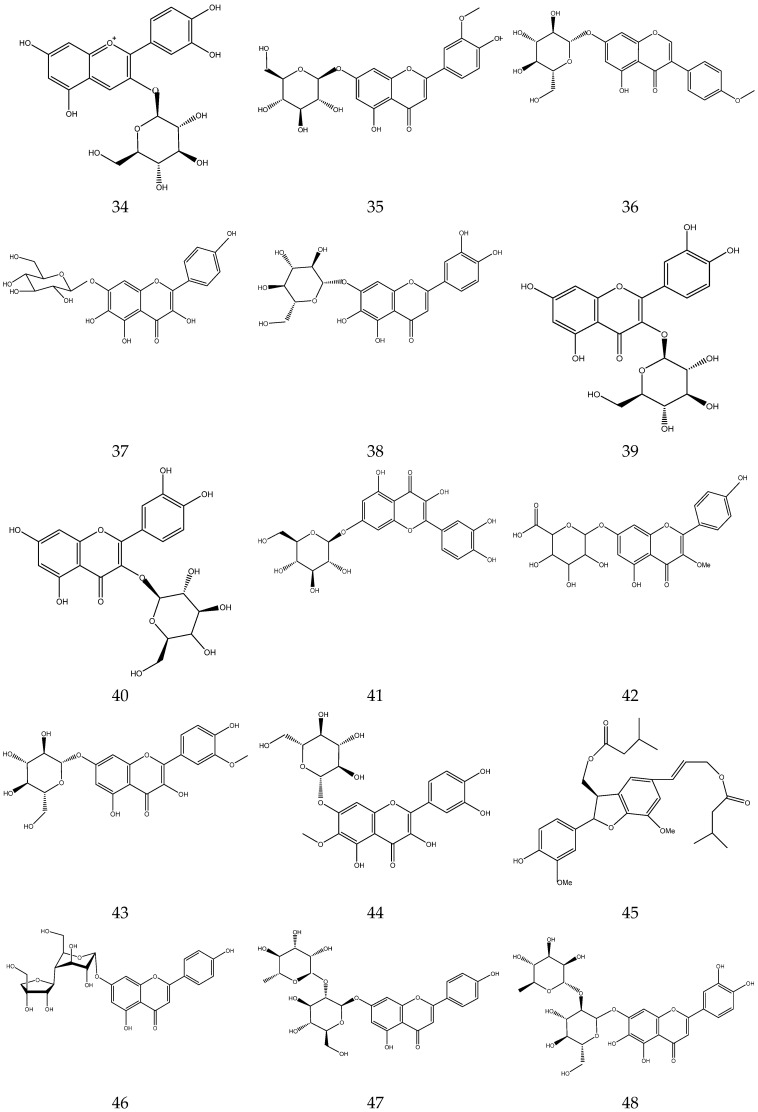

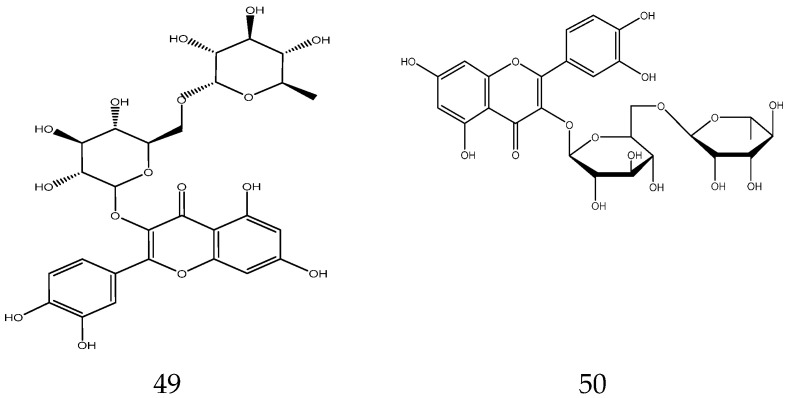
The chemical structures of flavonoids from Chamomile.

The flavonoids from Chamomile are shown in Table 3, and their chemical structures are shown in Figure 3.

#### 2.3.3. Coumarins

The parent nucleus of coumarin is benzopyrone. A total of 10 coumarins, including coumarin, 7-methoxycoumarin, esculetin, skimmin, daphnin, daphnetin, umbelliferone, scopoletin, isoscopoletin, and 3,4-Dihydrocoumarin, have been identified from Chamomile [33,34,38,43]. Li et al. established a method of simultaneous quantitative analysis for multi-components by single marker (QAMS) to determine the content of 7-methoxycoumarin, which uses an external standard method to determine apigenin, and uses a relative correction factor to determine 7-methoxycoumarin and other components [44].

**Figure 4:**
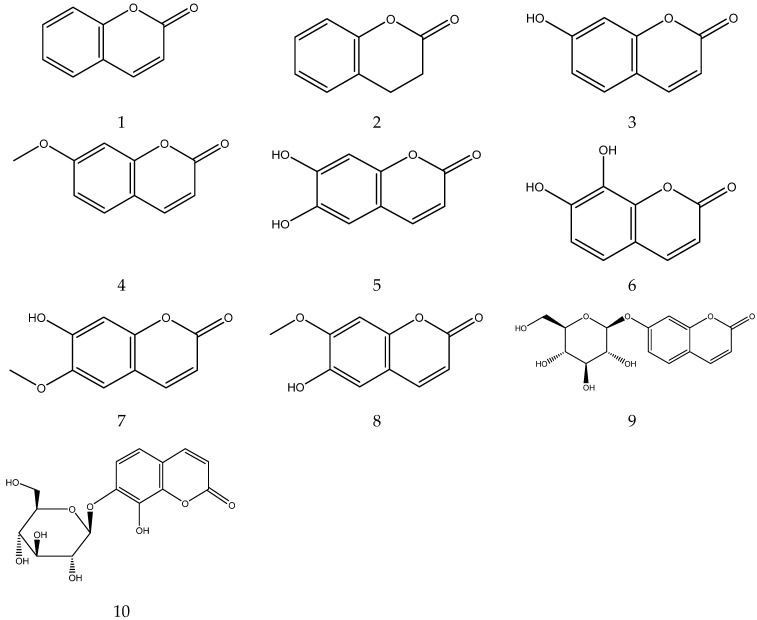
The chemical structures of coumarin from Chamomile.

The coumarins from chamomile are shown in Table 4 and their chemical structures are shown in Figure 4.

#### 2.3.4. Volatile Oil

The volatile oil of Chamomile has been prepared using a water distillation method, and its components have been identified using gas chromatography-mass spectrometry (GC-MS). A total of 102 components are reported in the volatile oil [33,34,36,37]. Volatile components, such as isopentyl isobutyrate, isobutyl isobutyrate, and others, have been reported to exhibit sedative and calming effects [45].

**Figure 5:**
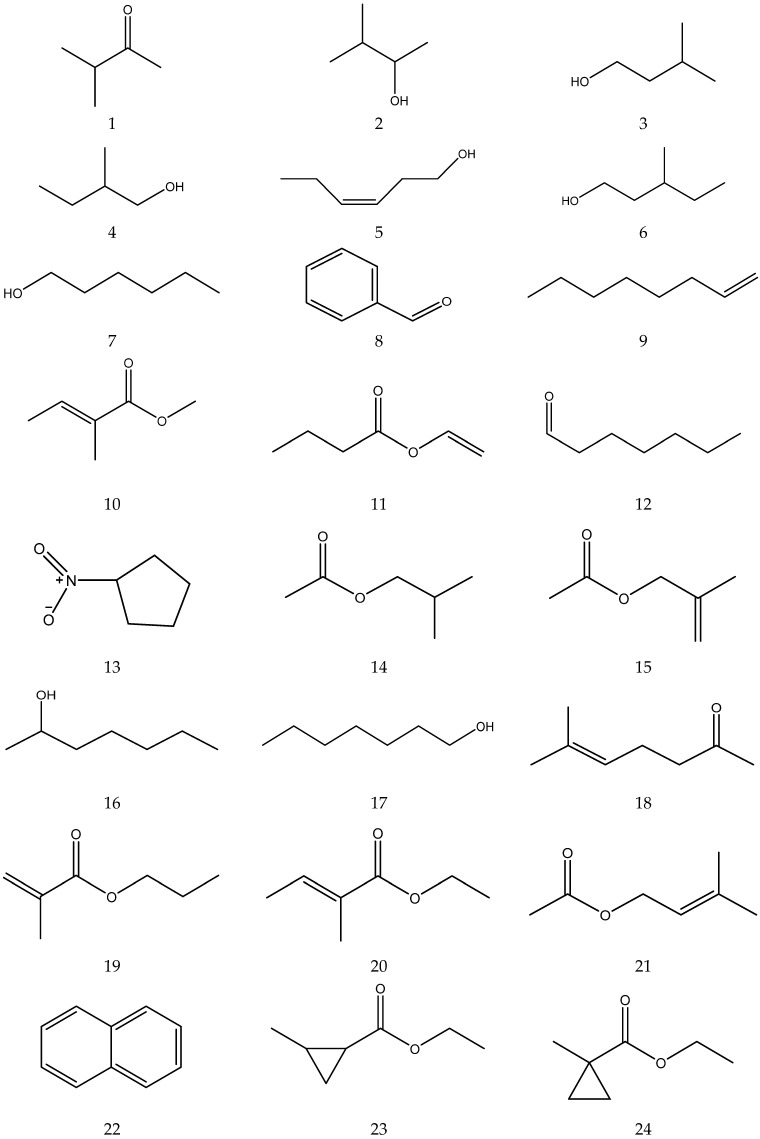

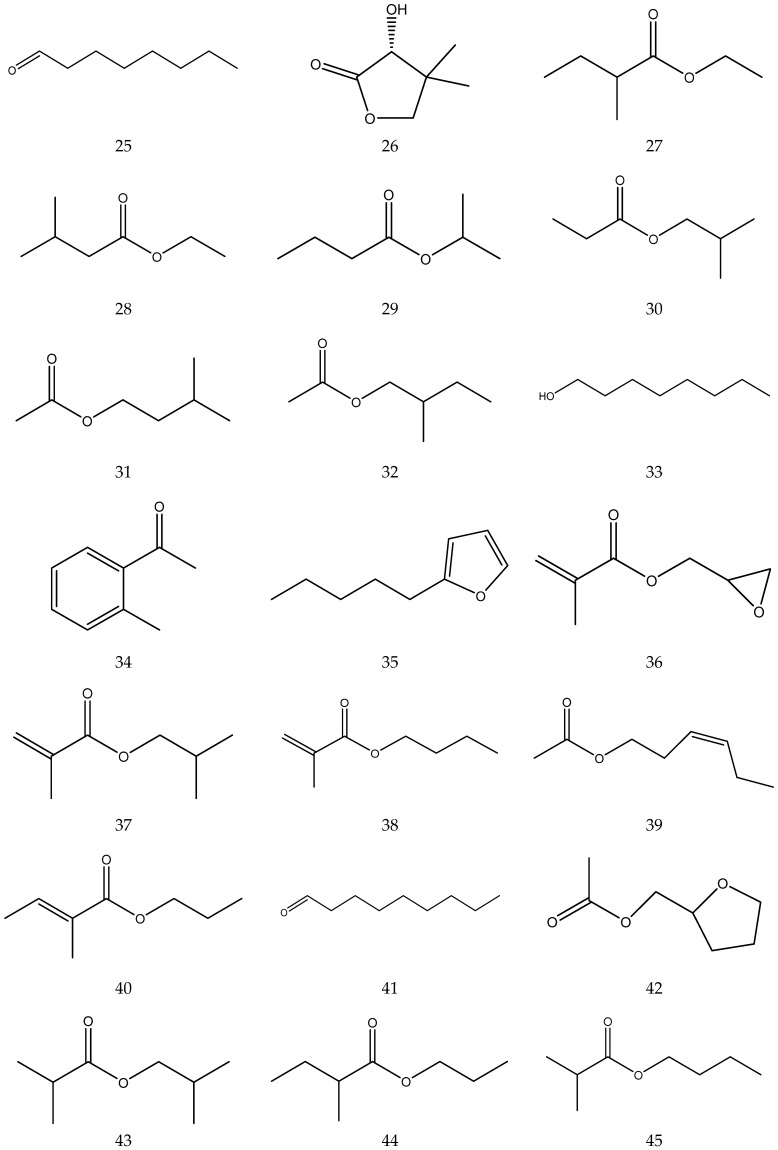

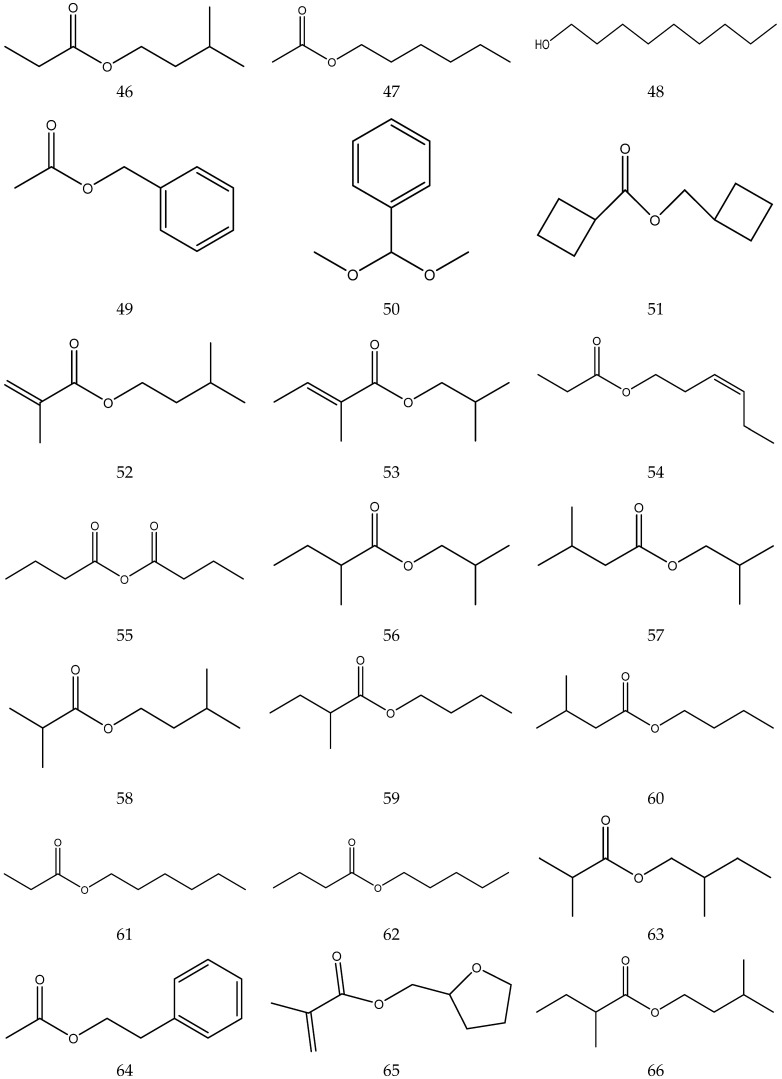

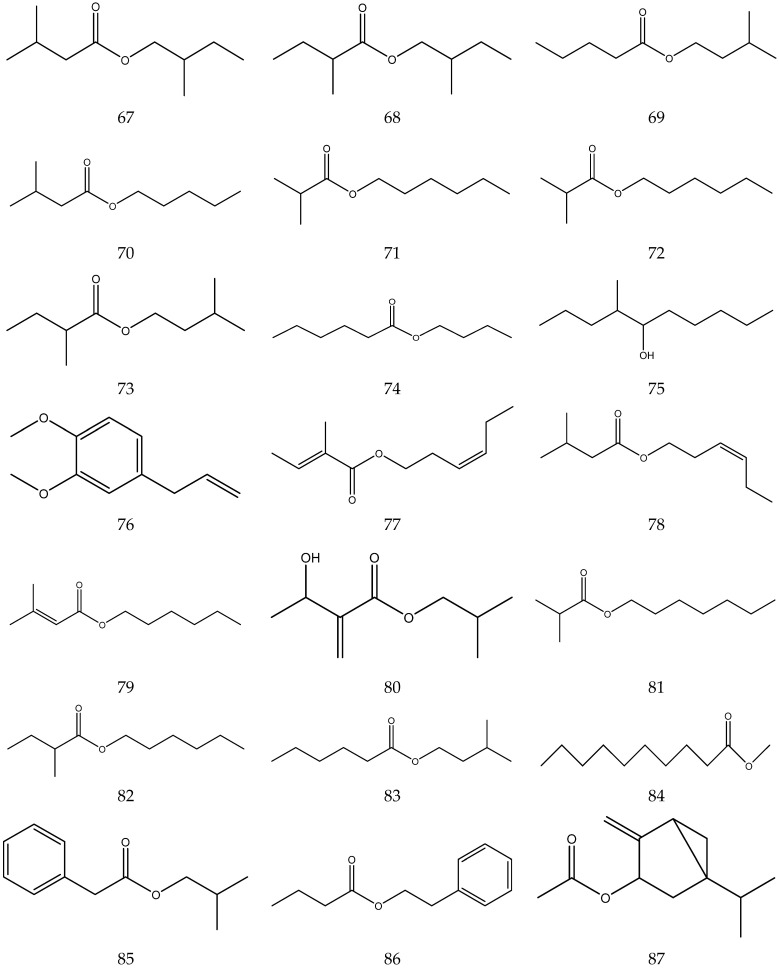

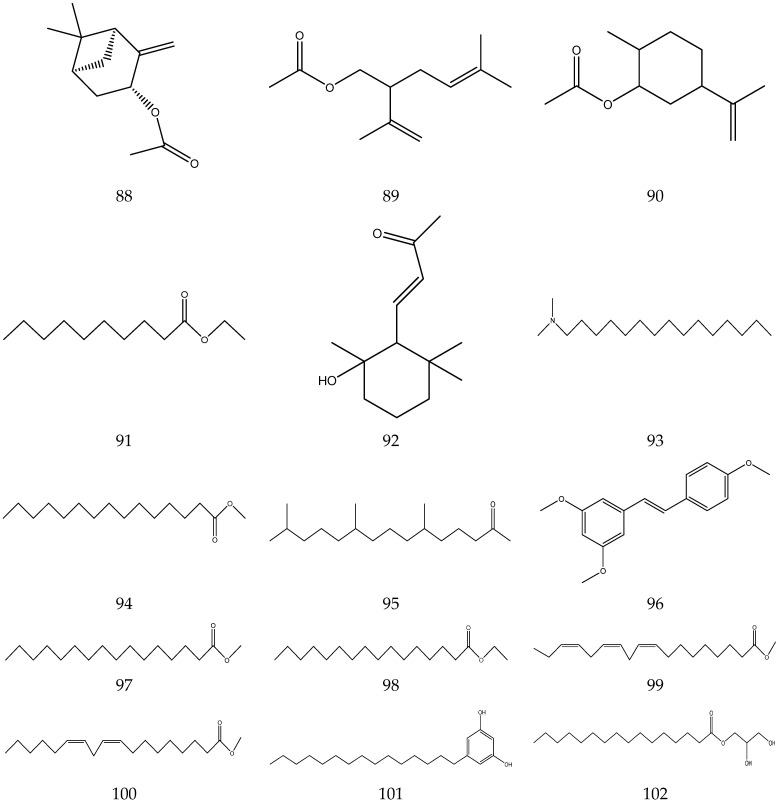
The chemical structures of volatile oil from Chamomile.

The chemical constituents of volatile oil from chamomile are shown in Table 5, and their chemical structures are shown in Figure 5.

#### 2.3.5. Monoterpenes

Monoterpenes contain two isoprene molecules. Three types of monoterpenes are present in Chamomile. They are (1) chain monoterpenes (e.g., ocimene, geraniol, citronellol) (2) monocyclic monoterpenes (menthol, and others), and (3) bicyclic monoterpenes such as bornanol. A total of 39 monoterpenes have been reported so far [33,34,35,36,37,38].

#### 2.3.6. Sesquiterpenes

Sesquiterpenes are polymerized from three molecules of isoprene. A total of 27 sesquiterpenes have been reported in chamomile so far [33,34,35,36,37,38]. Among them, *α*-bisabolol has anti-inflammatory activity, thereby protecting against acute liver injury caused by acetaminophen (APAP) [46]. 

#### 2.3.7. Diterpenes and Triterpenes

Diterpenes contain four isoprene units, whereas triterpenes contain six isoprene units. Up to now, two diterpenes (alcohol and phytanetriol) [33,36] and three triterpenes (oleanolic acid, taraxanol, and taraxasterol) have been reported in chamomile [7].

**Figure 6:**
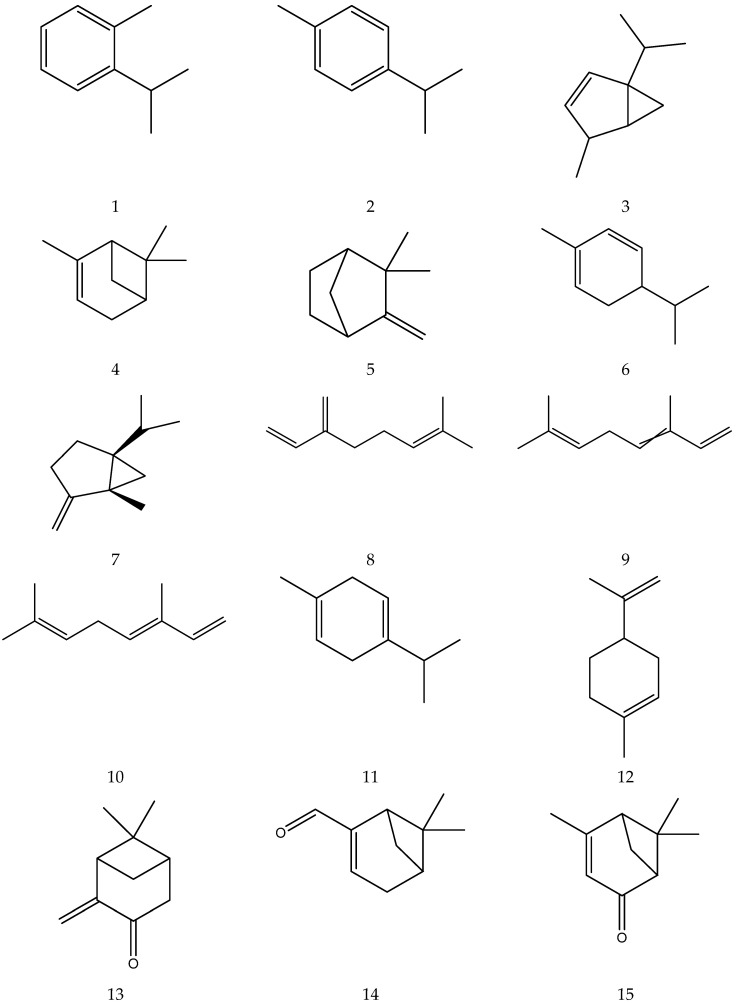

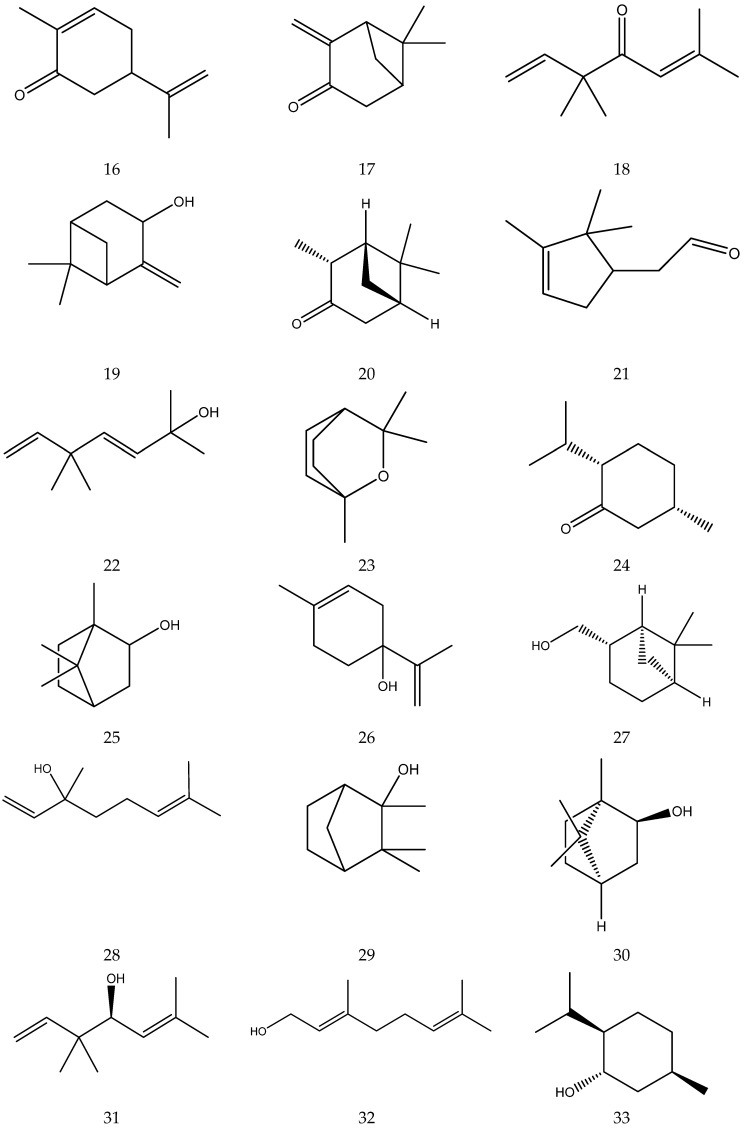

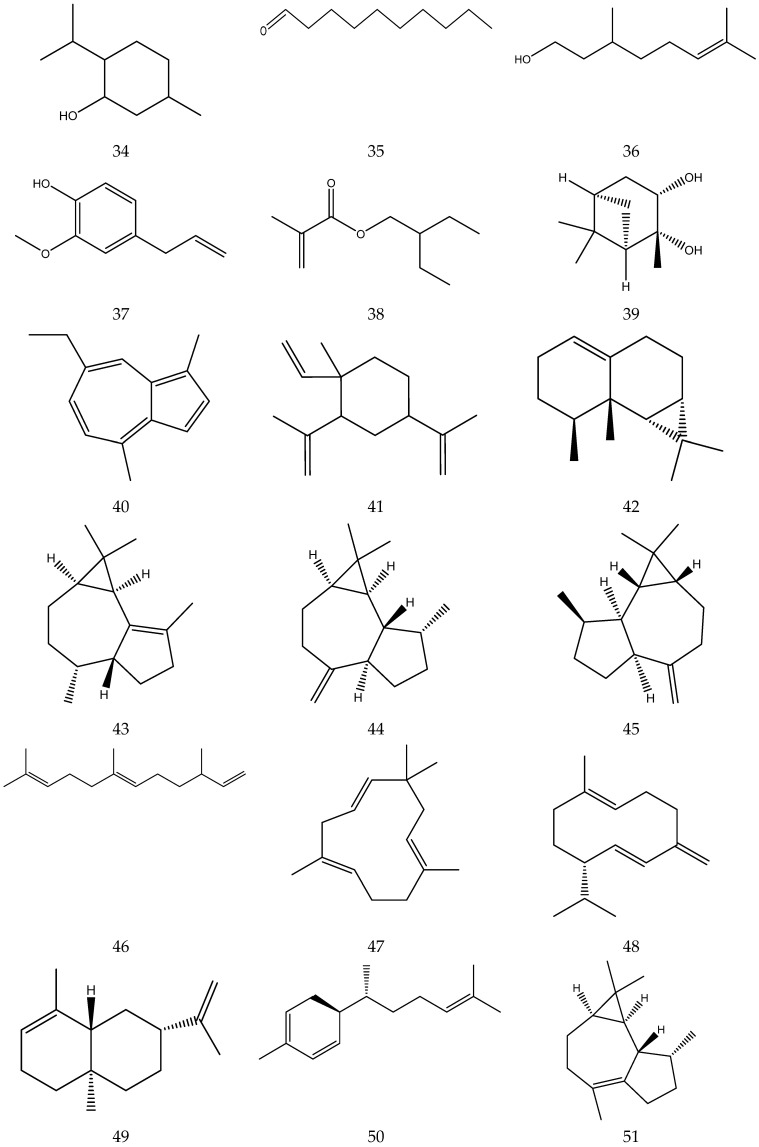

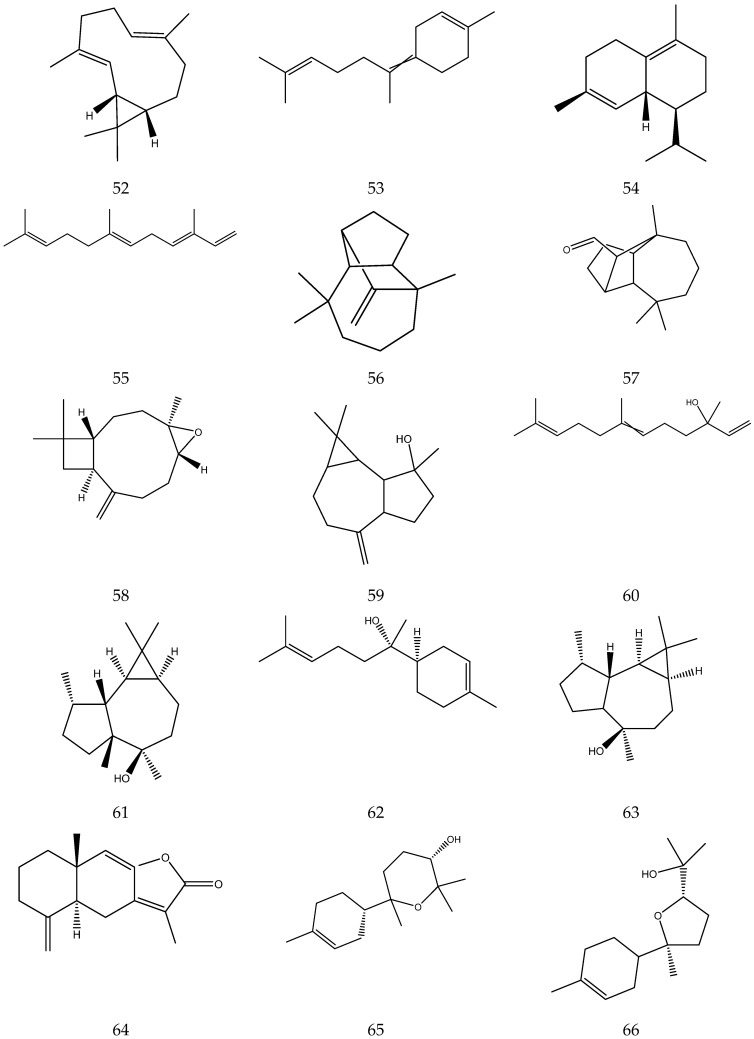

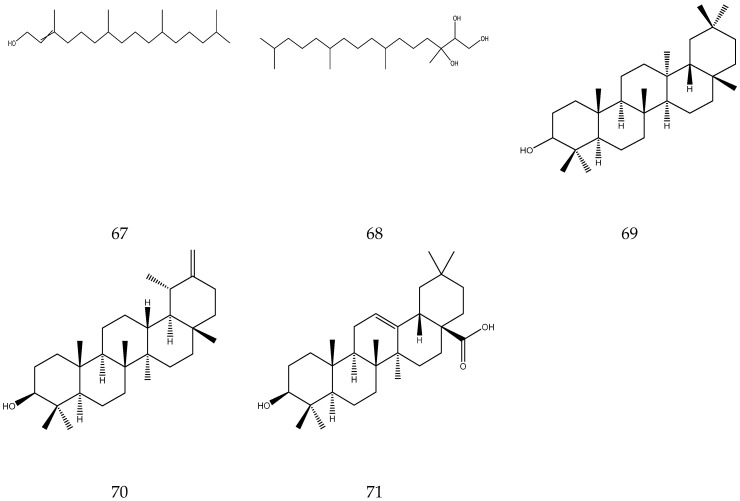
The chemical structures of terpenes from Chamomile.

The terpenes (monoterpenes, sesquiterpenes, diterpenes and triterpenes) from chamomile are shown in Table 6, and their chemical structures are shown in Figure 6.

#### 2.3.8. Other Phytochemicals

**Figure 7:**
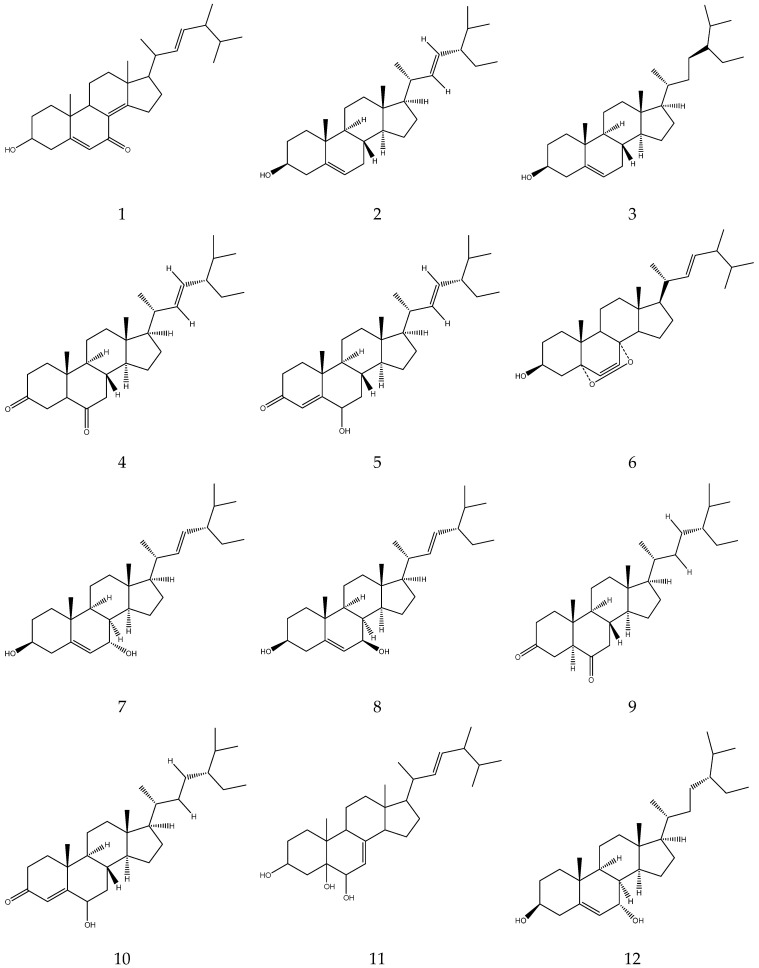

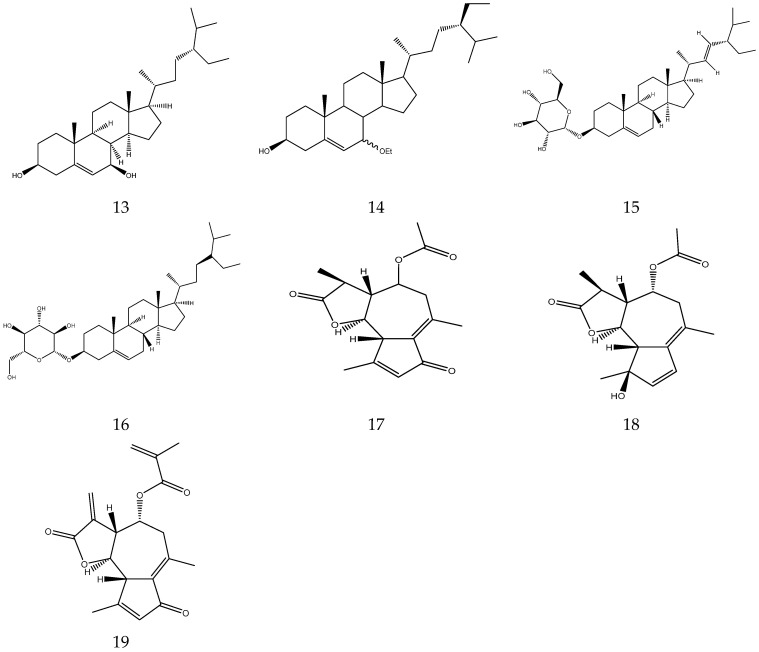
The chemical structures of sterols and guaiacolides from Chamomile.

Chamomile contains 1.29–3.25% polysaccharides. Upon hydrolysis they yield 45% D-galacturonic acid, 20.8% D-xylose, 12.2% D-galactose, 10.2% L-arabinose, 5.3% L-rhamnose and 2.3% D-glucose [47]. A total of 16 sterols (e.g., stigmasterol, taraxasterol) and three guaiacolides (guaianolide, matricin and matricarin) have so far been reported by Zhao [7] as shown in Table 7 and Figure 7 below.

L(+)-ascorbic acid, adenosine, phenyl propionic acids, benzodiazepines, γ-aminobutyric acid (GABA), choline, bitter substances, gums, amino acids, and (Z,E)-en-yn-dicycloether [7,33,34], as well as trace elements, such as Ca, Zn, Fe, Mg, Mn, Na, As, also exists in Chamomile [48,49], also exist in Chamomile.

## 3. Pharmacological Activities

### 3.1. Anticancer Activity

Glioma is one of the common intracranial malignant tumors with high incidence, rapid growth, high recurrence rate, high mortality and poor prognosis. α-Bisabolol, a fat soluble sesquiterpene compound that is widely found in Chamomile essential oil, has been proven to possess the potential to affect glioma. Yan et al. tested the effect of α-bisabolol on human brain glioblastoma cells (U251 and U87) using the scratch assay. Its effect on migration and invasion was investigated. Protein expression studies have been conducted using Western blot. α-Bisabolol inhibited gliobla stoma cell migration and invasion by down regulating central mucoepidermoid tumor (c-Met) [50]. α-Bisabolol oxide A and apigenin-7-β-D-glucoside, obtained from chamomile flowers and stems, are reported to inhibit the migration of Caco-2 colon cancer cells and deactivate the vascular epidemal growth factor receptor-2 (VEGFR2) angiogenic enzymes [51]. Apigenin is a flavonoid component of this plant, which also shows a certain anticancer effect on the liver cancer cells (Hep G2) and leukaemia cells (HL-60) [52]. Additionally, Srivastava et al. confirmed that apigenin 7-O-glycoside obtained from chamomile extract had a good anticancer effect, but its effect was lower than that of apigenin [53].

In vitro studies confirmed the antiproliferative effect of this plant on cervical cancer cells (HeLa) [54]. The anticancer activity is mediated through the Wnt pathway in colon tissue, down-regulating the expression levels of factors such as wingless integration-5A (Wnt5A), β-catenin, transfer cell factor 4 (Tcf4), and up-regulating the expression levels antigen presenting cell (APC) and GSK-3β [55]. Hydroalcoholic extracts of chamomile (dose-and time-dependent) have been reported to increase apoptosis and necrosis, decrease cell proliferation or migration, colonization, invasion and attachment in Michigan cancer foundation-7 (MCF-7) and MDA-MB-468 cell lines [56]. Chamomile fermented with *Lactobacillus plantarum* for 72 h showed selective cytotoxicity on cancer cells compared to normal cells (medical research council cell strain 5 (MRC-5)) [57].

### 3.2. Anti-Infective Activity

Chamomile volatile oil has shown an anti-infective effect on the growth of fungi and bacteria [58]. Furthermore, it effectively reduces the protease in mites and can be used to treat urticaria [59]. Mean corpsular hemoglobin-ampicillin 1 (MCh-AMP1), a natural peptide from the plant, has broad-spectrum antifungal activity against human pathogenic moulds and yeasts. It kills *Candida albicans* by increasing cell membrane permeability and inducing reactive oxygen species (ROS) production [60]. Shikov et al. reported that the minimun inhibitory concentration 90 (MIC90) and MIC50 of chamomile extract against *Helicobacter pylori* were 125 and 62.5 mg/mL, respectively. In addition, this extract controls the production of urease via modulating the morphology of *H. pylori* and fermentation capacity [61]. At present, many mouthwashes and sprays made with chamomile are used for oral bacteriostasis in clinical products, such as the White Gold Medal Compositae essence Product [62].

### 3.3. Anti-Inflammatory Activity

The flavonoids in chamomile are reported to be responsible for its anti-inflammatory effects. The possible mechanism involves the suppression of nuclear factor kappa beta (NF-κB)-driven transcription [63]. Yuan et al. reported the potent anti-inflammatory activity of Chamomile volatile oil in animal models mediated by inhibiting the production of inflammatory mediators (tumor necrosis factor alpha (TNF-α) and interleukin-1β (IL-1β)) [64]. Because of this activity, chamomile is often used to treat inflammatory diseases such as mammitis, colitis, dermatitis, cystitis, and conjunctivitis [12]. Chamomile Jinshui, an essential oil mainly composed of Chamomile, can effectively relieve prickly heat in children caused by sweat gathering around sweat glands in summer [16].

### 3.4. Antithrombotic Activity

Cardiovascular diseases (CVD) are one of the leading causes of death worldwide. Chamomile extract exhibits antithrombotic activity by prolonging coagulation and hemostasis time [65]. Luteolin in this plant prevents the development of oxidative stress in adenosine diphosphate (ADP)-induced carotid artery thrombosis in rats [66]. Bijak et al. reported that polyphenol-polysaccharide conjugate obtained from chamomile exerts an antithrombotic effect by reducing platelet aggregation [67]. Bas et al. discovered that half-maximal inhibitory concentrations (IC_50_) of water and butanol extracts on angiotensin-converting enzyme (ACE) were 1.292 mg/mL and 0.353 mg/mL, respectively [68]. All of the above findings further validate the antithrombotic activity of chamomile.

### 3.5. Antioxidant Activity

Volatile oil [69], polysaccharides [70,71] and total flavonoids [72,73] of Chamomile have been confirmed to scavenge 1,1-diphenyl-2-picrylhydrazyl (DPPH) and hydroxyl free radicals. The antioxidant effect is dose-dependent [74]. In addition, chamomile ethanol extract increases the activities of superoxide dismutase (SOD) and glutathione peroxidase (GSH-PX) and reduces the malondialdehyde (MDA) content in mice [75]. These findings provide a scientific basis for the antioxidant effect of Chamomile.

### 3.6. Hypoglycaemic Activity

Chamomile extract reduces fasting blood glucose levels in diabetic mice [75,76] and normal mice and improves glucose tolerance [77]. In addition, the extract antagonizes the effect of exogenous glucose [65]. Yang et al. reported the hypoglycaemic effect of total flavonoids in this plant occurs by reducing fibrinogen (FBG), glycated haemoglobin, glucose tolerance and glycated serum protein (GSP) levels in diabetic mice, and promoting glucose tolerance and insulin secretion [78]. Cemek et al. investigated the hypoglycemic effect of chamomile extract in streptozotocin (STZ)-diabetic rats. The results showed that it protected islet cells and reduced the oxidative stress associated with hyperglycaemia [79].

### 3.7. Antihypertensive Activity

Studies have found an antihypertensive activity of chamomile extract in essential hypertensive rats [80]. At the same time, another study found that chamomile extract did not affect the systolic blood pressure (SBP) and diastolic blood pressure (DBP) in normotensive rats, suggesting it has no toxic side effects in normal blood pressure regulation [81]. Luo et al. reported that the antihypertensive activity of chamomile extract in N-omega-nitro-L-arginine (L-NNA)-induced hypertensive rats is mediated by reducing angiotension Ⅱ (Ang Ⅱ) content and oxidative stress, and increasing SOD content [82].

### 3.8. Hypolipidaemic Activity

Hyperlipidaemia (HLP) refers to a metabolic disorder syndrome in which lipid components in plasma are abnormally dysregulated (increased serum total cholesterol (TC), triglyceride (TG), and low-density lipoprotein cholesterol (LDL-C), with decreased high-density lipoprotein cholesterol (HDL-C)). Chamomile is an effective blood lipid-lowering herb. Lan et al. reported that a chamomile alcohol extract played a role in lowering lipids, reducing TC, TG and LDL-C values and elevating HDL-C values in the blood of experimental hyperlipidemic rats [83]. A compound in fuzhuan tea, a popular compound tea containing chamomile with a mellow taste and no toxic side effects, can also treat HLP by regulating the above various sterol indicators [84].

### 3.9. Antiallergy Activity

As a conventional medicine, chamomile is frequently used to relieve various allergic symptoms. Antiallergic tea, as an example, has positive anti-allergy activity and cosmetology when it is drunk for a long time [21]. The antiallergic activity of chamomile aqueous extract (1.0 mg/mL) was reported by measuring the β-hexosaminidase (β-Hex) release in rat basophil leukemia (RBL-2H3) cells. This was suggested to inhibit the β-Hex release by 21.42% [85]. A chamomile methanol extract could restrain compound 48/80-induced allergic reactions. The effect was dose-dependent and mediated via decreased histamine release and NO levels from mast cells [86].

### 3.10. Antidepressant Activity

Essential oil aromatherapy is considered an alternative treatment for depression. Chamomile is an excellent reliever when patients with depression have physical and psychological discomfort [8]. Chamomile tea made from chamomile flower heads can effectively relieve depressive symptoms and the sleep status of postpartum women, which provides a new idea for treatment of depression [87]. Some pharmacological experiments have suggested the antidepressant activity of Chamomile [88]. For example, α-pinene contained in this plant elevated protein expression related to oxidative phosphorylation and mRNA expression of parvalbumin in rat brain, as determined by isobaric tag for relative and absolute quantitation (iTRAQ) and polymerase chain reaction (PCR) analysis [89].

### 3.11. Organoprotective Effect

Chamomile has protective effects on organs such as the liver, lung, kidney, and stomach, among others.

#### 3.11.1. Hepatoprotective and Pulmoprotective Effect

Chamomile flavonoids ameliorated mouse liver injury by restoring biochemical and molecular parameters in 1,2-Dimethyl hydrazine (DMH)-induced mice [90]. Zhang et al. ascertained that apigenin in this plant could treat APAP-induced liver injury by activating the adenosine monophosphate-activated protein kinase/GSK-3β (AMPK/GSK-3β) signaling pathway, promoting carnitine palmitoyl transferase 1A (CPT1A) activity and activating the nuclear factor erythroid 2-related factor 2 (Nrf2) antioxidant pathway [91]. Additionally, chamomile increases the total antioxidant capacity (TAC) and tissue transglutaminase (tTG) content in liver tissue, protecting against oxidative liver injury from paraquat (PQ) poisoning [92]. On the other hand, its extract protects oxidative lung damage from PQ poisoning, mainly by improving lipid peroxidation (LPO), SOD, glutathione peroxidase (GPx), and increasing the transporter associated with antigen processing (TAP) in plasma and lung tissue [93].

#### 3.11.2. Nephroprotective Effect

According to records in the “Baidu Yi Medicine Book”, chamomile is able to resolve the threat posed by kidney stones [7]. Modern pharmacological studies confirm this plant is an alternative therapy for gastric protection. For example, Salama et al. evaluated the protective effect of chamomile against cisplatin nephrotoxicity by intraperitoneal injection in rats. The study demonstrated that it reduces oxidative stress markers, corrects hypocalcemia caused by cisplatin nephrotoxicity, and inhibits glutamyltransferase activity [94]. In addition, its extract also inhibits phenomena such as glomerular fibrosis, improves renal tissue structure, and protects against renal tissue damage resulting from hypertension [82].

#### 3.11.3. Gastroprotective Effect

Chamomile is a promising gastroprotective herb to deal with stomach spasm, flatulence, stomachache, and decreased gastric secretion [95]. Its extract has shown antiulcer and antioxidant effects in ethanol-induced gastric mucosal injury in rats. Gastroprotective effects are mediated by reducing MDA levels, increasing GSH levels [96], protecting gastric sulfhydryl groups and the opposing effects of intracellular mediators such as free iron, hydrogen peroxide, and calcium [97].

### 3.12. Genitoprotective Effect

Chamomile extract improves reproductive function in polycystic ovary syndrome (PCOS) rats. Chamomile reduced the uterine and insulin resistance index, regulated sex hormones, leptin and blood lipids, and decreased inflammatory cells [77]. In addition, the interaction of chamomile with the GABA system can regulate luteinizing hormone (LH) secretion and increase dominant follicles for improving reproductive function in rats [98]. Soltani et al. performed surgical experiments on rats and treated the experimental group with chamomile extract. Histological characterization showed that the extract protected the testicular tissue from torsion/detorsion-induced damage by reducing MDA levels and inhibiting superoxide production [99]. Afrigan et al. injected formaldehyde and chamomile extract intraperitoneally into male Wistar rats to probe the hormonal status and sperm parameters of testicular tissue. It was found that this extract reduced the adverse effects of formaldehyde on the reproductive system in male rats [100].

### 3.13. Neuroprotective Effect

Chamomile has an excellent neuroprotective effect. Its extract restores scopolamine-decreased brain-derived neurotrophic factor (BDNF) expression, increases IL1β, and modulates cholinergic activity in the rat hippocampus [101]. It has been reported that a chamomile ethanol extract improves formaldehyde-induced memory impairment by reducing cell death and MDA content in the hippocampus, and increasing total antioxidant capacity [102]. Furthermore, Lim et al. found that apigenin in this plant inhibits H2O2-induced hippocampal cell (HT22) death [103]. Khan et al. reported the anti-Parkinson activity of chamomile extract by establishing an experimental animal model with chlorpromazine (CPZ). The extract showed vascular proliferation and increased the number of reactive glial cells [104].

### 3.14. Analgesic Activity

As early as hundreds of years ago, Chamomile was used as an analgesic remedy to relieve a variety of pains, such as arthralgia, stomach cramps, and neuralgia [13]. Nowadays, chamomile oil gel has been authenticated as an analgesic by reducing migraine pain without aura [105]. Saghafi et al. also reported the breast pain-relieving effect of chamomile (treated for 8 weeks) in patients using a visual analogue scale (VAS) and a breast pain scale (BPC) [106].

### 3.15. Antidiarrheal and Antispasmodic Activity

Chamomile is widely used in traditional Tunisian medicine and TCM against diarrhea and spasticity. In Germany, the extract of this plant is effective in treating children’s acute diarrhea by reducing symptoms and shortening the duration of disease [107]. Mehmood et al. reported the antidiarrheal and antispasmodic effects of chamomile using isolated rabbit jejunum. The chamomile extract activates K^+^ channels and reduces Ca^2+^ antagonism [108]. Hichem Sebai reported the beneficial effects of the extract in castor oil-induced diarrhea, which decreased MDA levels and antioxidant enzyme activity [109]. In addition, apigenin and apiin in Chamomile have a strong antispasmodic effect on smooth muscle [38].

### 3.16. Cosmetic Activity

Chamomile is useful in repairing sensitive skin, eliminating acne, and improving skin dehydration. It whitens the skin by inhibiting tyrosinase activity [110]. Therefore, it can be used as an ingredient in skincare products [38]. Ointments, creams and lotions containing chamomile active ingredients are used for the treatment of various skin infections and rashes in Europe and other places [111]. Chamomile Natural Milk Hand Soap, is widely popular for decontamination and sterilization, and can effectively moisturize the skin, enhance elasticity, and calm broken micro-blood vessels [112].

### 3.17. Other Activities

Chamomile alleviates muscle atrophy by suppressing muscle ring finger-1 (MuRF1) and increasing mitochondrial transcription factor A (TFAM), MyoD and myogenin-1 genes [113]. It relieves stiffness and pain in people suffering from knee osteoarthritis [114]. It also relieves general anxiety [115,116], with efficacy equivalent to conventional anti-anxiety drugs [117]. Its n-butanol extract showed a beneficial effect in relieving asthma in mice by reducing the eosinophils (EOS) and MDA levels and increasing IL-2, IL-10, IL-12 and SOD levels [118]. Studies report that Chamomile accelerates the healing of skin wounds by promoting fibroblast proliferation and migration [119]. Wan et al. indicated that apigenin in this plant inhibits transforming growth factor β1 (TGF-β1)-stimulated cardiac fibroblasts (CFs) differentiation and extracellular matrix (ECM) production by reducing microRNA-155-5p (miR-155-5p) expression, increasing c-Ski expression, and lowering Smad2/3 and p-Smad2/3 expression [120]. Studies report that chamomile extract abrogates withdrawal behavioural manifestations in morphine-dependent rats [121].

**Figure 8:**
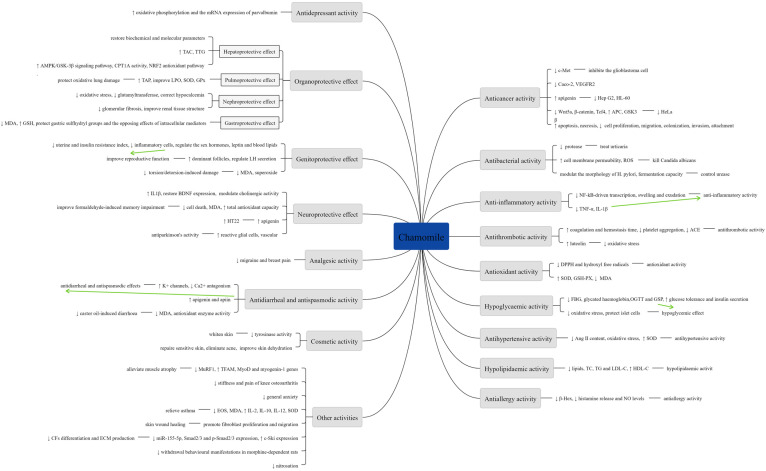
Summary of various pharmacological activities of Chamomile.

A summary of various pharmacological activities of Chamomile are shown in Figure 8.

## 4. Other Aspects

### 4.1. Adverse Reactions

According to records, the interaction between chamomile and some plants or drugs may cause adverse reactions. In theory, a combination of medicines that affect platelet aggregation may interfere with the effect of coagulation, thus increasing the risk of bleeding. Furthermore, high doses of Chamomile pose a teratogenic risk, affect the menstrual cycle, and cause vomiting [122]. Although this plant has antiallergic activity, a few studies have reported allergic reactions such as contact dermatitis and hypersensitivity, especially in people who are allergic to pollen or compositae [123,124]. There is a possibility to reduce follicular function and development, leading to premature birth in pregnant women when using chamomile [125,126].

### 4.2. Toxicity Studies

There have been reports of chamomile as a potential carrier of Clostridium botulinum spores [127]. Kalantari et al. reported the genotoxicity of Chamomile in mice using ultra-viable micronucleus assay of reticulocytes [128]. However, Wang et al. reported that the aqueous and alcohol extracts of this plant are safe for mice. It has been reported that the maximum tolerated dose of aqueous and alcohol extracts are 535 and 425 times higher than the usual adult dose in a clinic [129]. So far, only a few studies have been carried out to evaluate its toxicity, and more studies are required to confirm the safety of consuming chamomile.

### 4.3. Clinical Preparation

Chamomile has long been known as a medicinal and aromatic plant in Europe, the United States, Japan and other countries. At present, due to its multiple medicinal effects, many chamomile-containing herbal medicinal products have been developed, such as chamomile ointment, cream and lotion [111]. However, there are relatively few preparations in clinical products. Clinical preparations of this plant are mainly used in clinical research in the form of a mouth rinse [130,131]. For example, the White Gold Medal Compositae essence Gargle, which was listed in Japan in 2002, is able to be applied to oral bacteriostasis, clean care, and relieve oral discomfort [62]. In addition, three kinds of chamomile preparations can be retrieved from the database of Chinese patent medicine prescriptions “https://db.yaozh.com/chufang (accessed on 17 November 2022)”, including Fufang Munizi granules, Zukamu capsules and Zukamu granules [4]. The U.S. National Drug Code also lists more than two hundred kinds of preparations containing this plant, such as Allergies (pellet), SleepCalm (spray), OHM Fever Relief (spray), and Melatonin Pro (tablet) [6]

### 4.4. Quality Control Studies

High Performance Liquid Chromatography (HPLC) is a standard method for identifying and quantifying the components in herbs to assess their quality. Furthermore, evaluating chamomile quality is crucial to reproducing its clinical efficacy. You et al. reported the HPLC method for the simultaneous determination of luteolin-7-O-β-D glucoside and apigenin 7-O-β-D glucoside in chamomile extract and displayed the linear range of luteolin-7-O-β-D glucoside and apigenin-7-O-β-D glucoside was 5.11~409.00 and 12.56~1005.00 μg/mL, respectively [132]. Lan et al. reported the UPLC method to determine the content of apigenin-7-glucoside in chamomile, and the content was found to be 5.2 and 5.8 mg/g, in two batches [133]. Wu et al. adopted this method to quantify α-bisabolol, and the concentration of α-bisabolol in MC was 0.045 mg/mL, whereas it was not detected in CN [134]. Gas chromatography also plays a vital role in the quality control of pharmaceuticals. Zhao et al. reported a gas chromatographic method to determine the volatile oil components in this plant. *α*-Bisabolol, α-bisabolol oxide A and α-bisabolol oxide B were present in volatile oil of MC, whereas CN volatile oil contained only α-bisabolol oxide A. Therefore, the comprehensive quality evaluation of MC and CN could be qualitatively and quantitatively based on the content of bisabolol or the presence or absence of *α*-bisabolol oxide B [95].

Total saponins content in Chamomile was determined by utlraviolet (UV) spectrophotometry, and the detected content was 8.02% [135]. Jing et al. used a flame atomic absorption spectrometric method to measure the content of trace elements (Ca, Zn, Fe, Mg, Mn, and Na). The contents of Ca, Zn, Fe, Mg, Mn and Na were 2.016, 0.032, 0.684, 2.380, 0.044 and 1.235 mg/g, respectively [48]. In addition, Xin et al. reported the presence of arsenic in this plant using atomic fluorescence spectroscopy [49]. A thin-layer chromatography method can be used to identify luteolin in chamomile [136]. QAMS is a research tool for multi-component quality control, which is used to solve the bottleneck problem when the control substance is scarce and expensive in the qualitative evaluation of herbal medicine [137]. Li et al. utilized QAMS to determine cynaroside, apigenin-7-glucoside, 7-methoxycoumarin, luteolin and apigenin in chamomile. There was no significant difference in the contents calculated using either this method or an external standard method [44]. Han et al. reported a method in which polyamide was used as a stationary phase and microemulsion as a developing agent to identify above five compounds. This method is better than thin layer chromatography using silica gel [138].

## 5. Discussion and Conclusions

A lot of research has been carried out on chamomile in recent years. This article reviews the latest research progress on this plant, including botanical characteristics, traditional uses, chemical constituents, pharmacological effects, and quality evaluation. A total of 301 compounds have been reported in Chamomile, including 26 organic acids, 50 flavonoids, 10 coumarins, 102 volatile oil constituents, 39 monoterpenes, 27 sesquiterpenes, 2 diterpenes, 3 triterpenes, 16 sterols, 6 polysaccharides, 3 guaiacolides, 7 trace elements and 10 other components. Flavonoids represented by apigenin have significant anti-inflammatory effects. Esters in volatile oils have sedative and anxiolytic effects. α-Bisabolol in sesquiterpenes can protect against APAP-induced acute liver injury. Chamomile also plays anticancer, anti-infective, antioxidant, hypoglycaemic, hypotensive, hypolipidaemic, antiallergic, antidepressant, organ protective, genitoprotective, and neuroprotective effects. In addition, chamomile is used as an ingredient in skin whitening products and relieves muscle atrophy, speeds up skin wound healing, and treats asthma. There are many scientific studies on it, but there are still numerous research gaps. For example, more toxicity tests should be carried out to confirm the safe use of chamomile. Its clinical effects depend on chemical composition and the amount of active components. HPLC is an effective analytical method for identifying and quantifying components in herbs. The content of luteolin-7-O-glucoside, apigenin-7-O-glucoside, chlorogenic acid, apigenin, apigenin-7-glucose, *α*-bisabolol and other substances have been quantified using various HPLC methods. In addition, volatile components such as *α*-bisabolol and *α*-bisabolol oxide B have been identified and quantified using gas chromatography. This method is useful to differentiate MC and CN.

In summary, chamomile is a widely used herb in the traditional medicine of Greece, Rome, China, Germany and other countries. It has great economic value due to its numerous pharmacological effects and wide uses. This paper summarizes its various aspects systematically. The information in this review will be a good scholarly resource for its further development and utilization. However, there is a need for further research to provide concrete scientific evidence and validate the medicinal uses of chamomile.

## Figures and Tables

**Table 1:** Traditional uses of chamomile.

No.	The Name or Origin of Traditional Uses	Main Components	Usage	Countries	Reference
1	Chamomile tea	*Matricaria chamomilla* L.	Clear away heat, eyesight, hypotensive and soothe the nerves and sleep	Turkey, China, Germany and other places	[7,18,25]
2	Honey chamomile tea	*Matricaria chamomilla* L., *Citrus limon* (L.) Osbeck, honey	Lower the level of high blood pressure, detoxify, and fight inflammation.	China	[22]
3	Medicinal Aroma	*Matricaria chamomilla* L., *Rosa rugosa* Thunb., *Lavandula angustifolia* Mill., *Lindera benzoin* (L.) Blume, *Citrus medica* L., *Salvia japonica* Thunb., *Nardostachys jatamansi* (D.Don) DC., *Melissa officinalis* L., *Pogostemon cablin* (Blanco) Benth.	Soothes the nerves	China	[26]
5	Qingdan Capsules	*Matricaria chamomilla* L., *Curcuma aromatica* Salisb., *Taraxacum mongolicum* Hand.-Mazz., *Gardenia jasminoides* J.Ellis, *Artemisia capillaris* Thunb., *Paeonia lactiflora* Pall., *Lysimachia christinae* Hance, *Poria cocos* (Schw.) Wolf., *Citrus sinensis* (L.) Osbeck	Clears gallbladder	China	[27]
6	Anti-itch bath	*Matricaria chamomilla* L., *Allium sativum* L., Squalane	Anti-itch	China	[17]
7	Anti-inflammatory bath	*Matricaria chamomilla* L., *Ligusticum striatum* DC., *Angelica sinensis* (Oliv.) Diels, *Citrus* × *aurantium* L.	Anti-inflammation	China	[17]
8	Chinese herbal bath	Matricaria chamomilla L., *Ligusticum striatum* DC., *Angelica sinensis* (Oliv.) Diels, *Adenophora stricta* Miq., *Citrus sinensis* (L.) Osbeck	Treats rheumatoid arthritis	Japan	[28]
9	Sunscreen skin care products	*Matricaria chamomilla* L., *Plumeria alba* L., *Mosla chinensis* Maxim., *Rheum palmatum* L., *Glycyrrhiza uralensis* Fisch., *Mentha canadensis* L.	Sun protection	China	[29]
10	Facial soap	*Matricaria chamomilla* L., *Citrus sinensis* (L.) Osbeck, *Angelica sinensis* (Oliv.) Diels, *Paeonia* × *suffruticosa* Andrews, *Paeonia lactiflora* Pall., almond oil	Improves dull and rough skin	Japan	[30]
11	Skin repair lotion	*Matricaria chamomilla* L., *Portulaca oleracea* L., *Glycyrrhiza uralensis* Fisch., *Centella asiatica* (L.) Urb.	Relieves skin irritation	China	[31]
12	Antiviral traditional Chinese medicine composition	*Matricaria chamomilla* L., *Lonicera japonica* Thunb., *Fagopyrum dibotrys* (D. Don) Hara, *Houttuynia cordata* Thunb., *Prunella vulgaris* L., *Morus alba* L., *Dendranthema morifolium* (Ramat.) Tzvelev, *Perilla frutescens* (L.) Britton, *Pogostemon cablin* (Blanco) Benth., *Mentha canadensis* L., Radix Puerariae, *Glycyrrhiza uralensis* Fisch., *Zingiber officinale* Roscoe, *Ziziphus jujuba* Mill.	Antiviral or prevents viral diseases	China	[32]

**Table 2:** Organic acids from Chamomile.

Categories	No	Name	Molecular Formula	Relative Molecular Mass	References
Primary metabolites	1	Palmitic acid	C_16_H_32_O_2_	256.42	[33]
	2	Linoleic acid	C_18_H_32_O_2_	280.45	[33]
	3	Oleic acid	C_18_H_34_O_2_	282.46	[33]
	4	Stearic acid	C_18_H_36_O_2_	284.48	[33]
Secondary metabolites	5	Isobutyric acid	C_4_H_8_O_2_	88.11	[36]
	6	Tiglic acid	C_5_H_8_O_2_	100.12	[36]
	7	Isovaleric acid	C_5_H_10_O_2_	102.13	[36]
	8	D,L-2-Methylbutyric acid	C_5_H_10_O_2_	102.13	[36]
	9	4-Hydroxybenzoic acid	C_7_H_6_O_3_	140.11	[35]
	10	Cinnamic acid	C_9_H_8_O_2_	149.02	[35]
	11	Anisic acid	C_8_H_8_O_3_	152.15	[33]
	12	Nonanoic acid	C_9_H_18_O_2_	158.24	[36]
	13	Vanillic acid	C_8_H_8_O_4_	168.15	[33]
	14	Decanoic acid	C_10_H_20_O_2_	172.26	[36]
	15	Caffeic acid	C_9_H_8_O_4_	179.04	[34]
	16	Quinic acid	C_7_H_12_O_6_	191.06	[35]
	17	Ferulic acid	C_10_H_10_O_4_	193.05	[35]
	18	Galacturonic acid	C_6_H_10_O_7_	194.14	[33]
	19	Syringic acid	C_9_H_10_O_5_	198.17	[33]
	20	Chlorogein acid	C_16_H_18_O_9_	353.09	[34]
	21	Neochlorogenic acid	C_16_H_18_O_9_	353.09	[34]
	22	Cryptochlorogenic acid	C_16_H_18_O_9_	354.31	[38]
	23	Isochlorogenic acid B	C_25_H_24_O_12_	516.40	[37]
	24	Isochlorogenic acid A	C_25_H_24_O_12_	516.45	[38]
	25	Isochlorogenic acid C	C_25_H_24_O_12_	516.46	[35]
	26	1,5-Dicaffeoylquinic acid	C_25_H_24_O_12_	517.13	[35]

**Table 3:** Flavonoids from Chamomile.

No	Name	Molecular Formula	Relative Molecular Mass	References
1	Apigenin	C15H10O5	269.05	[37]
2	Galangin	C_15_H_10_O_5_	270.24	[35]
3	Naringenin	C15H12O5	272.25	[33]
4	Luteolin	C_15_H_10_O_6_	286.24	[33]
5	Kaempferol	C_15_H_10_O_6_	286.24	[35]
6	Hispidulin	C16H12O6	300.26	[35]
7	Hydroxygenkwanin	C16H12O6	300.26	[35]
8	Chrysoeriol	C16H12O6	300.26	[33]
9	Quercetin	C_15_H_10_O_7_	302.23	[35]
10	Ermanin	C_17_H_14_O_6_	314.29	[35]
11	3-O-Methylquercetin	C16H12O7	316.26	[33]
12	3-Methylquercetin/Isorhamnetin	C16H12O7	316.26	[33]
13	6-Methoxykaempfero	C_16_H_12_O_7_	316.26	[35]
14	Eupafolin	C16H12O7	316.26	[35]
15	Eupalitin	C17H14O7	330.29	[33]
16	Jaceosidin	C17H14O7	330.29	[35]
17	Patuletin	C16H12O8	332.26	[33]
18	Esculin	C15H16O9	339.07	[34]
19	Eupatoletin	C_17_H_14_O_8_	346.29	[33]
20	Spinacetin	C17H14O8	346.29	[33]
21	Axillarin	C17H14O8	346.29	[33]
22	Scopolin	C16H18O9	355.10	[34]
23	Jaceidin	C18H16O8	360.31	[33]
24	Chrysosplenol D	C18H16O8	360.31	[33]
25	4′,5-Di-hydroxy-3,3′,6,7-tetra-methoxy-flavone	C_19_H_18_O_8_	374.34	[33]
26	Chrysosplenetin B	C_19_H_18_O_8_	374.34	[33]
27	Cosmosiin	C_21_H_20_O_10_	431.10	[33]
28	Sophoricoside	C21H20O10	433.11	[34]
29	Luteolin-4′-β-glucoside	C21H20O11	448.38	[40]
30	Cynaroside	C_21_H_20_O_11_	448.38	[40]
31	Quercitrin	C21H20O11	448.38	[33]
32	Astragalin	C21H20O11	448.38	[33]
33	Cynaroside	C21H20O11	449.11	[33]
34	Cyanidin-3-O-glucoside	C21H21O11	449.11	[34]
35	Chrysoeriol-β-glucoside	C_22_H_22_O_11_	462.40	[33]
36	Pratensein-7-O-glucoside	C22H22O11	463.12	[34]
37	7-O-(β-D-glucopyranosyl)-galactin	C_21_H_20_O_12_	464.20	[7]
38	6-Hydroxy-luteolin-7-glucoside	C_21_H_20_O_12_	464.38	[33]
39	Quercetin-3-glucoside	C21H20O12	464.38	[33]
40	Hyperoside	C21H20O12	464.38	[33]
41	Quercimeritrin	C21H20O12	466.39	[33]
42	Isokaempferol-7-O-glucuronide	C_22_H_20_O_12_	476.21	[7]
43	Isorhamnetin-7-O-β-glucoside	C_22_H_22_O_12_	478.41	[33]
44	Patulitrin	C22H22O13	494.40	[33]
45	Dimerized coniferyl isovalerate	C_30_H_38_O_8_	526.30	[7]
46	Apiin	C26H28O14	564.49	[33]
47	Rhoifolin	C27H30O14	578.52	[34]
48	Luteolin-7-O-β-rutinoside	C_27_H_30_O_15_	594.52	[33]
49	Quercetin-3-O-rutinoside	C_27_H_30_O_16_	610.52	[33]
50	Rutin	C_27_H_30_O_16_	610.52	[33]

**Table 4:** Coumarins from Chamomile.

No	Name	Molecular Formula	Relative Molecular Mass	References
1	Coumarin	C_9_H_6_O_2_	146.14	[33]
2	3,4-Dihydrocoumarin	C_9_H_8_O_2_	148.16	[38]
3	Umbelliferone	C_9_H_6_O_3_	162.14	[43]
4	7-Methoxycoumarin	C_10_H_8_O_3_	177.05	[34]
5	Esculetin	C_9_H_6_O_4_	178.14	[33]
6	Daphnetin	C_9_H_6_O_4_	178.14	[43]
7	Scopoletin	C_10_H_8_O_4_	192.17	[33]
8	Isoscopoletin	C_10_H_8_O_4_	192.17	[33]
9	Skimmin	C_15_H_16_O_8_	324.28	[43]
10	Daphnin	C_15_H_16_O_9_	340.28	[43]

**Table 5:** The chemical constituents of volatile oil from Chamomile.

No	Name	Molecular Formula	Relative Molecular Mass	References
1	3-Methyl-2-butanone	C_5_H_10_O	86.13	[36]
2	3-Methyl-2-butanol	C_5_H_12_O	88.15	[36]
3	Isoamyl alcohol	C_5_H_12_O	88.15	[36]
4	2-Methyl-1-butanol	C_5_H_12_O	88.15	[36]
5	Leaf alcohol	C_6_H_12_O	100.16	[36]
6	3-Methyl-1-pentanol	C_6_H_14_O	102.17	[36]
7	Hexyl alcohol	C_6_H_14_O	102.17	[36]
8	Benzaldehyde	C_7_H_6_O	106.12	[36]
9	1-Octene	C_8_H_16_	112.21	[36]
10	Methyl tiglate	C_6_H_10_O_2_	114.14	[36]
11	Vinyl butyrate	C_6_H_10_O_2_	114.14	[33]
12	Heptaldehyde	C_7_H_14_O	114.19	[36]
13	Cyclopentane, nitro-	C_5_H_9_NO_2_	115.13	[33]
14	Isobutyl acetate	C_6_H_12_O_2_	116.16	[36]
15	Methallyl acetate	C_6_H_12_O_2_	116.16	[36]
16	2-Heptanol	C_7_H_16_O	116.20	[36]
17	Heptylalcohol	C_7_H_16_O	116.20	[36]
18	Methylheptenone	C_8_H_14_O	126.20	[36]
19	Propyl methacrylate	C_7_H_12_O_2_	128.17	[36]
20	Ethyl tiglate	C_7_H_12_O_2_	128.17	[36]
21	Prenyl acetate	C_7_H_12_O_2_	128.17	[36]
22	Naphthalene	C_10_H_8_	128.17	[36]
23	Ethyl 2-methylcyclopropanecarboxylate	C_7_H_12_O_2_	128.17	[33]
24	Ethyl 1-methylcyclopropanecarboxylate	C_7_H_12_O_2_	128.17	[33]
25	Octanal	C_8_H_16_O	128.21	[36]
26	Dihydro-3-hydroxy-4,4-dimethyl-, (R)-2(3H)-Furanone	C_6_H_10_O_3_	130.14	[36]
27	Ethyl 2-methylbutyrate	C_7_H_14_O_2_	130.18	[35]
28	Ethyl isovalerate/Ethyl 3-methylbutanoate	C_7_H_14_O_2_	130.18	[35]
29	Isopropyl butyrate	C_7_H_14_O_2_	130.18	[36]
30	Isobutyl propionate	C_7_H_14_O_2_	130.18	[36]
31	Isoamyl acetate	C_7_H_14_O_2_	130.18	[36]
32	2-Methylbutyl acetate	C_7_H_14_O_2_	130.18	[36]
33	1-Octanol	C_8_H_18_O	130.23	[36]
34	2′-Methylacetophenone	C_9_H_10_O	134.18	[36]
35	2-Pentylfuran	C_9_H_14_O	138.21	[36]
36	2-Propenoic acid, 2-methyl-, oxiranylmethyl ester	C_7_H_10_O_3_	142.15	[33]
37	Isobutyl methacrylate	C_8_H_14_O_2_	142.20	[33]
38	Butyl methacrylate	C_8_H_14_O_2_	142.20	[36]
39	Leaf acetate	C_8_H_14_O_2_	142.20	[36]
40	Propyl tiglate	C_8_H_14_O_2_	142.20	[36]
41	1-Nonanal	C_9_H_18_O	142.24	[36]
42	2-Furanmethanol, tetrahydro-, acetate	C_7_H_12_O_3_	144.17	[33]
43	Isobutyl isobutyrate	C_8_H_16_O_2_	144.21	[33]
44	Propyl 2-methylbutanoate	C_8_H_16_O_2_	144.21	[36]
45	Butyl isobutyrate	C_8_H_16_O_2_	144.21	[36]
46	Isoamyl propionate	C_8_H_16_O_2_	144.21	[36]
47	Hexyl acetate	C_8_H_16_O_2_	144.21	[36]
48	1-Nonanol	C_9_H_20_O	144.25	[36]
49	Benzyl acetate	C_9_H_10_O_2_	150.17	[36]
50	Benzaldehyde dimethyl acetal	C_9_H_12_O_2_	152.19	[36]
51	Cyclobutanecarboxylic acid, cyclobutyl ester	C_9_H_14_O_2_	154.21	[33]
52	Iso-amyl methacrylae	C_9_H_16_O_2_	156.22	[36]
53	Isobutyl tiglate	C_9_H_16_O_2_	156.22	[36]
54	Cis-3-hexexnyl propionate	C_9_H_16_O_2_	156.22	[36]
55	Butyric anhydride	C_8_H_14_O_3_	158.19	[33]
56	Isobutyl 2-methylbutyrate	C_9_H_18_O_2_	158.24	[36]
57	Isobutyl isovalerate	C_9_H_18_O_2_	158.24	[36]
58	Isopentyl isobutyrate	C_9_H_18_O_2_	158.24	[36]
59	2-Methyl-butanoic acid butyl ester	C_9_H_18_O_2_	158.24	[37]
60	Butyl isovalerate	C_9_H_18_O_2_	158.24	[36]
61	Hexyl propionate	C_9_H_18_O_2_	158.24	[36]
62	Amyl butyrate	C_9_H_18_O_2_	158.24	[36]
63	2-Methylbutyl isobutyrate	C_9_H_18_O_2_	158.24	[33]
64	Phenethyl acetate	C_10_H_12_O_2_	164.20	[36]
65	2-Propenoic acid, 2-methyl-,(tetrahydro-2-furanyl) methyl ester	C_9_H_14_O_3_	170.21	[33]
66	Isoamyl 2-methyl butyrate	C_10_H_20_O_2_	172.26	[36]
67	2-Methylbutyl isovalerate	C_10_H_20_O_2_	172.26	[36]
68	2-Methylbutyl 2-methylbutyrate	C_10_H_20_O_2_	172.26	[36]
69	Pentanoic acid, 3-methylbutyl ester	C_10_H_20_O_2_	172.26	[36]
70	3-Methyl-butanoic aci pentyl ester	C_10_H_20_O_2_	172.26	[36]
71	Hexyl isobutyrate	C_10_H_20_O_2_	172.26	[36]
72	Hexyl isobutyrate	C_10_H_20_O_2_	172.26	[36]
73	Butanoic acid, 2-methyl-, 3-methylbutyl ester	C_10_H_20_O_2_	172.26	[33]
74	Hexanoic acid,2-methylpropyl ester	C_10_H_20_O_2_	172.26	[33]
75	4-Methyl-5-decanol	C_11_H_24_O	172.31	[33]
76	Methyl eugenol	C_11_H_14_O_2_	178.23	[36]
77	Cis-3-Hexenyl tiglate	C_11_H_18_O_2_	182.26	[36]
78	3-Methyl-, 3-hexenyl ester, (Z)-Butanoic acid	C_11_H_20_O_2_	184.27	[36]
79	2-Butenoic acid,3-methyl-, hexyl ester	C_11_H_20_O_2_	184.28	[36]
80	Isobutyl 3-hydroxy-2-methylenebutanoate	C_10_H_18_O_3_	186.25	[33]
81	Propanoic acid, 2-methyl-, heptyl ester	C_11_H_22_O_2_	186.29	[36]
82	Hexyl 2-methylbutyrate	C_11_H_22_O_2_	186.29	[36]
83	Isopentyl hexanoate	C_11_H_22_O_2_	186.29	[33]
84	Decanoic acid, methyl ester	C_11_H_22_O_2_	186.29	[33]
85	Isobutyl phenylacetate	C_12_H_16_O_2_	192.25	[36]
86	Phenethyl butyrate	C_12_H_16_O_2_	192.25	[36]
87	Sabinylacetate	C_12_H_18_O_2_	194.27	[36]
88	(−)-Trans-pinocarvyl acetate	C_12_H_18_O_2_	194.27	[33]
89	Lavandulyl acetate	C_12_H_20_O_2_	196.29	[36]
90	(R)-Dihydrcarvyl acetate	C_12_H_20_O_2_	196.29	[36]
91	Decanoic acid, ethyl ester	C_12_H_24_O_2_	200.32	[33]
92	3-Buten-2-one, 4-(2-hydroxy-2,6,6-trimethylcyclohexyl)	C_13_H_22_	210.31	[33]
93	1-Pentadecanamine, N,N-dimethyl	C_17_H_37_N	255.48	[33]
94	Pentadecanoic acid, methyl ester	C_16_H_32_O_2_	256.42	[33]
95	Perhydrofarnesyl acetone	C_18_H_36_O	268.48	[36]
96	3,4′,5-Trimethoxy-*trans*-stilbene	C_17_H_18_O_3_	269.05	[34]
97	Hexadecanoic acid, methyl ester	C_17_H_34_O_2_	270.45	[37]
98	Hexadecanoic acid, ethyl ester	C_18_H_36_O_2_	284.48	[37]
99	9,12,15-Octadecatrienoic acid, methyl ester, (Z,Z,Z)-	C_19_H_32_O_2_	292.46	[33]
100	9,12-Octadecadienoic acid (Z,Z)-, methyl ester	C_19_H_34_O_2_	294.47	[33]
101	5-Pentadecylresorcinol	C_21_H_36_O_2_	320.51	[33]
102	Monopalmitin	C_19_H_38_O_4_	330.50	[33]

**Table 6:** Terpenes from chamomile.

Categories	No	Name	Molecular Formula	Relative Molecular Mass	References
Monoterpenes	1	O-Cymene	C_10_H_14_	134.22	[36]
	2	P-Cymene	C_10_H_14_	134.22	[35]
	3	α-Thujene	C_10_H_16_	136.23	[36]
	4	α-Pinene	C_10_H_16_	136.23	[36]
	5	Camphene	C_10_H_16_	136.23	[36]
	6	3-Cyclohexadiene, 2-methyl-5-(1-methylethyl)-1	C_10_H_16_	136.23	[36]
	7	4(10)-Thujene, (1R,5R)-(+)-(8CI)/d-SabiNene	C_10_H_16_	136.23	[36]
	8	Myrcene	C_10_H_16_	136.23	[36]
	9	(Z)-Ocimene	C_10_H_16_	136.23	[36]
	10	(E)-3,7-Dimethylocta-1,3,6-triene	C_10_H_16_	136.23	[36]
	11	γ-Terpinene	C_10_H_16_	136.23	[36]
	12	Dipentene	C_10_H_16_	136.23	[37]
	13	Pinocarvone	C_10_H_14_O	150.22	[36]
	14	Myrtenal	C_10_H_14_O	150.22	[36]
	15	(−)-Verbnone	C_10_H_14_O	150.22	[36]
	16	Carvone	C_10_H_14_O	150.22	[36]
	17	2(10) -Pinen-3-one	C_10_H_14_O	150.22	[33]
	18	3,3,6-Trimethyl-1,5-heptadien-4-one	C_10_H_16_O	152.23	[36]
	19	Pinocarveol	C_10_H_16_O	152.23	[36]
	20	Isocamphopinone	C_10_H_16_O	152.23	[36]
	21	3-Cyclopentene-1-acetaldehyde,2,2,3-trimethyl-	C_10_H_16_O	152.23	[33]
	22	Yomogi alcohol	C_10_H_18_O	154.25	[36]
	23	Cineole	C_10_H_18_O	154.25	[36]
	24	5-Methyl-2-(1-methylethyl)-, cis-Cyclohexanonene	C_10_H_18_O	154.25	[36]
	25	D,L-2-Bornanol	C_10_H_18_O	154.25	[36]
	26	4-Carvomenthenol	C_10_H_18_O	154.25	[36]
	27	(−)-Trans-Myrtanol	C_10_H_18_O	154.25	[36]
	28	1,6-Octadien-3-ol,3,7-dimethyl-	C_10_H_18_O	154.25	[33]
	29	Camphene hydrate	C_10_H_18_O	154.25	[33]
	30	Bicyclo[2.2.1]heptan-2-ol, 1,7,7-trimethyl-, (1S-endo)-	C_10_H_18_O	154.25	[33]
	31	Artemisia alcohol	C_10_H_18_O	154.25	[37]
	32	Geraniol	C_10_H_18_O	154.25	[37]
	33	D-Isomenthol	C_10_H_20_O	156.27	[36]
	34	D,L-Menthol	C_10_H_20_O	156.27	[36]
	35	Decanal	C_10_H_20_O	156.27	[36]
	36	Citronellol	C_10_H_20_O	156.27	[37]
	37	Eugenol	C_10_H_12_O_2_	164.20	[36]
	38	2-Propenoic acid, 2-methyl-, 2-ethylbutyl ester	C_10_H_18_O_2_	170.25	[36]
	39	(1R,2R,3S,5R)-(−)-2,3-Pinanediol	C_10_H_18_O_2_	170.25	[33]
Sesquiterpenes	40	Chamazulen	C_14_H_16_	184.28	[36]
	41	β-Elemene	C_15_H_24_	204.35	[36]
	42	(+)-Calarene	C_15_H_24_	204.35	[36]
	43	(−)-α-Gurjunene	C_15_H_24_	204.35	[36]
	44	(+)-Aromadendrene	C_15_H_24_	204.35	[36]
	45	(−)-Allaromadendrene	C_15_H_24_	204.35	[36]
	46	(E)-β-Farnesene	C_15_H_24_	204.35	[36]
	47	Humulene	C_15_H_24_	204.35	[36]
	48	Germacrene D	C_15_H_24_	204.35	[36]
	49	α-Selinene	C_15_H_24_	204.35	[36]
	50	α-Zingiberene	C_15_H_24_	204.35	[36]
	51	(+)-Ledene	C_15_H_24_	204.35	[36]
	52	(+)-Bicyclogermacrene	C_15_H_24_	204.35	[36]
	53	β-Bisabolene	C_15_H_24_	204.35	[36]
	54	(+)-*δ*-Cadnene	C_15_H_24_	204.35	[36]
	55	α-Farnesene	C_15_H_24_	204.35	[36]
	56	D-Longifolene	C_15_H_24_	204.35	[33]
	57	Longifolenaldehyde	C_15_H_24_O	220.35	[33]
	58	Cayophyllene oxide	C_15_H_24_O	220.35	[37]
	59	Spathulenol	C_15_H_24_O	220.40	[36]
	60	Nerolidol	C_15_H_26_O	222.37	[36]
	61	Himbaccol	C_15_H_26_O	222.37	[36]
	62	α-Bisabolol	C_15_H_26_O	222.37	[36]
	63	Epiglobulol	C_15_H_26_O	222.37	[33]
	64	Atractylenolide Ⅰ	C_15_H_18_O_2_	231.14	[34]
	65	α-Bisabolol oxide A	C_15_H_26_O_2_	238.37	[36]
	66	α-Bisabolol oxide B	C_15_H_26_O_2_	238.37	[36]
Diterpenes	67	Plant alcohol	C_20_H_40_O	296.53	[33]
	68	Phytanetriol	C_20_H_42_O_3_	330.55	[36]
Triterpenes	69	Taraxerol	C_30_H_50_O	426.72	[7]
	70	Taraxasterol	C_30_H_50_O	426.72	[7]
	71	Oleanolic Acid	C_30_H_48_O_3_	456.71	[7]

**Table 7:** Sterols and guaiacolides from Chamomile.

Categories	No	Name	Molecular Formula	Relative Molecular Mass	References
Sterols	1	(22E,24R)-3β-Hydroxyergosta-5,8,22-trien-7-one	C_28_H_42_O_2_	410.69	[7]
	2	Stigmasterol	C_29_H_48_O	412.70	[7]
	3	β-sitosterol	C_29_H_50_O	414.72	[7]
	4	Stigmasteran-22-ene-3,6-dione	C_29_H_46_O_2_	426.70	[7]
	5	6β-Hydroxystigmaster-4,22-dien-3-one	C_29_H_46_O_2_	426.70	[7]
	6	3β-Hydroxy-6,22-diene-5α,8β-cyclodioxyergosterol	C_28_H_44_O_3_	428.69	[7]
	7	7α-Hydroxy-β-stigmasterol	C_29_H_48_O_2_	428.70	[7]
	8	7β-Hydroxy-β-stigmasterol	C_29_H_48_O_2_	428.70	[7]
	9	5α-Stigmasteran-3,6-dione	C_29_H_48_O_2_	428.70	[7]
	10	6-Hydroxystigmaster-4-en-3-one	C_29_H_48_O_2_	428.70	[7]
	11	7,22-Diene-3,5,6-trihydroxy-ergosterol	C_28_H_46_O_3_	430.69	[7]
	12	7α-Hydroxy-β-sitosterol	C_29_H_50_O_2_	430.72	[7]
	13	7β-Hydroxy-β-sitosterol	C_29_H_50_O_2_	430.72	[7]
	14	3β-Hydroxy-7α-hydroxyethyl-24β-ethyl-cholest-5-ene	C_31_H_54_O_2_	458.72	[7]
	15	Stigmasterol 3-O-β-D glucoside	C_35_H_58_O_6_	574.84	[7]
	16	Daucosterol	C_35_H_60_O_6_	576.86	[7]
Guaiacolides	17	Matricarin	C_17_H_20_O_5_	304.34	[7]
	18	Matricin	C_17_H_22_O_5_	306.35	[7]
	19	Guaianolide	C_20_H_20_O_5_	340.20	[7]

## Abbreviations

Abbreviations	Full name
TCM	Traditional Chinese medicine
MC	*Matricaria chamomilia* L.
CN	*Chamaemelum nobile* (L.) All.
4-MUO	4-Methylumbelliferone oleate
LPS	lipopolysaccharide
BV2	a microglia
GSK-3β/Nrf2	Glycogen Synthase Kinase 3β/Nuclear factor E2 related factor 2
QAMS	quantitative analysis for multi-components by single marker
GC-MS	gas chromatography-mass spectrometry
APAP	acetaminophen
MyoD	myogenic differentiation
GABA	γ-aminobutyric acid
c-Met	central mucoepidermoid tumor
Caco-2	a colon cancer cell
VEGFR2	vascular epidemal growth factor receptor-2
Hep G2	a liver cancer cell
HL-60	a leukaemia cell
HeLa	a cervical cancer cell
Wnt5A	wingless integration-5A
Tcf4	transfer cell factor 4
APC	antigen presenting cell
MCF-7	michigan cancer foundation-7
MDA-MB-468	a triple-negative breast cancer cell line
MRC-5	medical research council cell strain 5
MCh-AMP1	Mean corpsular hemoglobin—ampicillin 1
ROS	reactive oxygen species
MIC90	minimun inhibitory concentration 90
NF-κB	nuclear factor kappa beta
TNF-α	tumor necrosis factor alpha
IL-1β	interleukin-1β
CVD	cardiovascular disease
ADP	adenosine diphosphate
IC_50_	half-maximal inhibitory concentrations
ACE	angiotensin-converting enzyme
DPPH	the 1,1-diphenyl-2-picrylhydrazyl
SOD	superoxide dismutase
GSH-PX	glutathione peroxidase
MDA	malondialdehyde
FBG	fibrinogen
GSP	glycated serum protein
STZ	streptozotocin
SBP	systolic blood pressure
DBP	diastolic blood pressure
L-NNA	N-omega-nitro-L-arginine
Ang Ⅱ	angiotension Ⅱ
HLP	hyperlipidaemia
TC	serum total cholesterol
TG	triglyceride
LDL-C	low-density lipoprotein cholesterol
HDL-C	high-density lipoprotein cholesterol
β-Hex	β-hexosaminidase
RBL-2H3	rat basophil leukemia
iTRAQ	isobaric tag for relative and absolute quantitation
PCR	polymerase chain reaction
DMH	1,2-Dimethyl hydrazine
TAC	total antioxidant capacity
tTG	tissue transglutaminase
PQ	paraquat
AMPK/GSK-3β	adenosine monophosphate-activated protein kinase/glycogen synthase kinase 3β
CPT1A	carnitine palmitoyl transferase 1A
Nrf2	nuclear factor erythroid 2-related factor 2
LPO	lipid peroxidation
GPx	glutathione peroxidase
TAP	transporter associated with antigen processing
PCOS	polycystic ovary syndrome
LH	luteinizing hormone
BDNF	brain-derived neurotrophic factor
HT22	a hippocampal cell
CPZ	chlorpromazine
VAS	visual analogue scale
BPC	breast pain scale
MuRF1	muscle ring finger-1
TFAM	mitochondrial transcription factor A
EOS	eosinophils
TGF-β1	transforming growth factor β1
CFs	cardiac fibroblasts
ECM	extracellular matrix
miR-155-5p	microRNA-155-5p
HPLC	High Performance Liquid Chromatography
UV	Utlraviolet
